# Identifying psychiatric manifestations in schizophrenia and depression from audio-visual behavioural indicators through a machine-learning approach

**DOI:** 10.1038/s41537-022-00287-z

**Published:** 2022-11-07

**Authors:** Shihao Xu, Zixu Yang, Debsubhra Chakraborty, Yi Han Victoria Chua, Serenella Tolomeo, Stefan Winkler, Michel Birnbaum, Bhing-Leet Tan, Jimmy Lee, Justin Dauwels

**Affiliations:** 1 School of Electrical and Electronic Engineering, Nanyang Technological University, Singapore, Singapore; 2 Institute of Mental Health, Singapore, Singapore; 3 School of Social Science, Nanyang Technological University, Singapore, Singapore; 4 Department of Psychology, National University of Singapore, Singapore, Singapore; 5 School of Computing, National University of Singapore, Singapore, Singapore; 6 Mindsigns Health, Singapore, Singapore; 7 Lee Kong Chian School of Medicine, Nanyang Technological University, Singapore, Singapore; 8 Faculty of Electrical Engineering, Mathematics, and Computer Science, Delft University of Technology, Delft, Netherlands

**Keywords:** Human behaviour, Biomarkers, Schizophrenia

## Abstract

Schizophrenia (SCZ) and depression (MDD) are two chronic mental disorders that seriously affect the quality of life of millions of people worldwide. We aim to develop machine-learning methods with objective linguistic, speech, facial, and motor behavioral cues to reliably predict the severity of psychopathology or cognitive function, and distinguish diagnosis groups. We collected and analyzed the speech, facial expressions, and body movement recordings of 228 participants (103 SCZ, 50 MDD, and 75 healthy controls) from two separate studies. We created an ensemble machine-learning pipeline and achieved a balanced accuracy of 75.3% for classifying the total score of negative symptoms, 75.6% for the composite score of cognitive deficits, and 73.6% for the total score of general psychiatric symptoms in the mixed sample containing all three diagnostic groups. The proposed system is also able to differentiate between MDD and SCZ with a balanced accuracy of 84.7% and differentiate patients with SCZ or MDD from healthy controls with a balanced accuracy of 82.3%. These results suggest that machine-learning models leveraging audio-visual characteristics can help diagnose, assess, and monitor patients with schizophrenia and depression.

## Introduction

Schizophrenia (SCZ) and depression (MDD) are two of the top 15 chronic mental disorders with severe impact on the people affected^1^. SCZ is a chronic and disabling disorder, characterized by positive (e.g., delusions and hallucinations), negative (e.g., anhedonia, asociality, avolition, affective blunting, and alogia), and cognitive (e.g., attention, memory, and problem solving) symptom. Recent studies suggest that negative symptoms may not be unique to SCZ as previously thought, as those symptoms have been observed in people with MDD and other mood disorders^2,3^. Similarly, cognitive deficits in people with MDD have become a clinically relevant target for treatment^4^. While positive symptoms are more readily identified and managed with effective medications, negative symptoms and cognitive impairments are often overlooked, less responsive to pharmacological interventions, and more closely associated with poor functional outcomes, resulting in a diminished quality of life for patients^5–7^.

In clinical practice today, the manuals for diagnosing mental disorders and psychometric tools are considered the gold standard for diagnosing and assessing mental illnesses. However, these tools rely on the interviewer’s experience; as a consequence, they introduce a degree of subjectivity, are resource-intensive, and offer limited information concerning the temporal and spatial dynamics underlying clinical symptoms and manifestations^8^. The data-driven approach can help us understand different diseases, better identify them, and save costs. These methods can be used as a form of prescreening and can support the diagnosis. In this study, we design an ensemble learning pipeline to measure thousands of behavioral traits, providing new insights into the behavioral changes in mental disorders.

Digital phenotyping, defined as the moment-by-moment quantification of the individual-level human phenotype in situ using data from personal digital devices^9^, offers an innovative lens to observe behaviors in naturalistic and longitudinal settings^10^. This approach also fits naturally into the NIMH’s Research Domain Criteria (RDoC) framework that suggests new ways of classifying mental disorders based on dimensions of observable behaviors and neurobiological measures^11^. Several implementations of digital phenotyping have been designed, guided by the RDoC, to quantify behaviors associated with mental illnesses objectively^12,13^. Along similar lines, many studies that analyze audio and visual data of patients with SCZ have demonstrated abnormalities in language^14–16^, speech^17–21^, facial expressions^21–23^, and motor^24–26^ behaviors. Similar studies of patients with MDD have identified abnormalities in verbal^27–30^ and nonverbal behaviors^31–36^, facial expressions^34,37,38^, and body movement^39–41^ associated with MDD. This stream of the literature suggests that digital phenotyping is a promising avenue toward objective behavioral measures for characterizing mental disorders.

Recent findings suggest that vocal and facial characteristics of patients with SCZ are associated with blunted affect and alogia^42,43^ . However, it remains unclear whether the behavioral phenotyping fueled by machine learning allows us to accurately predict the overall severity of negative symptoms and other psychiatric symptoms. Clinical evaluation typically requires combining multiple heterogeneous sources of information, but the potential of combining multiple modalities for diagnosis and measuring the psychiatric state of patients with SCZ has not been investigated so far. For MDD, most machine-learning-based studies aim to detect depression and predict depressive symptoms automatically. No studies have tried to predict negative symptoms in patients with MDD using behavioral cues. For both SCZ and MDD, machine-learning pipelines with audio-visual behavioral cues for detecting neurocognitive deficits have not yet been developed. Moreover, except for Lott and Kliper^44,45^, none of the existing studies consider differential diagnostic groups using machine-learning techniques; instead, they are limited to a single psychiatric disorder.

In preliminary studies, we observed that language, acoustic, conversation, and body movement biomarkers can be used to predict several subscales of the 16-item Negative Symptom Assessment Scale (NSA-16) for SCZ and differentiate SCZ from healthy controls (HCs), respectively^15,19,20,24^. We also found that by combining verbal and acoustic features, it is possible to predict several NSA-16 subscales (e.g., NSA-2: Restricted speech quantity and NSA-15: Reduced expressive gestures) for SCZ and MDD and to differentiate MDD and SCZ from HCs^46^. In this study, we explore the prediction of multiple types of symptom domains using various behavior features in both SCZ and MDD. Specifically, our aims were to: (1) extend earlier studies on multi-modality behavioral analysis by combining a plethora of modalities, including verbal, nonverbal (acoustic, prosodic, articulate, phonetic, and conversational features), facial (emotional, facial, and eye-movement features), and body movement cues; (2) extend existing studies on automated detection of negative symptoms to a series of symptoms: negative, cognitive, and general psychiatric symptoms; (3) develop a modular machine-learning pipeline such that additional behavioral cues can readily be integrated into the pipeline without having to redesign the entire system, and (4) investigate whether the proposed digital phenotype models are consistent and stable across different time points and different samples, which constitutes a first small step towards automated longitudinal follow-up of negative (and other) symptoms in psychiatric patients.

## Results

### Data collection

**Fig. 1:**
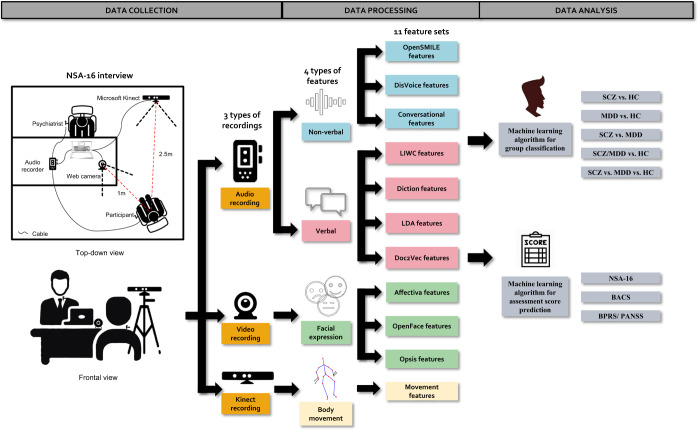
Diagram of the analysis pipeline. The audio-visual recordings were collected during the Negative Symptom Assessment-16 interview. We extracted 11 feature sets from the recordings. These feature sets were utilized to classify different groups of participants and predict the symptom severity.

We collected the audio-visual datasets from two studies (see Fig. 1): Study-A was a longitudinal study with three study visits including 54 SCZ and 26 HCs, and Study-B was a cross-sectional study including 49 SCZ, 50 MDD, and 49 HCs.

In both studies, all participants were first assessed for negative symptoms using the NSA-16^47^ in a semi-structured interview. We derived four symptom domain factors, i.e., restricted speech (NSA-RS), poor quality of speech (NSA-PQ), affective blunting (NSA-AB), and amotivation (NSA-AM), from factor analysis; we analyzed the weighted factor scores as well as the total score (NSA-Total)^48^.

We leveraged the Brief Assessment of Cognition in Schizophrenia (BACS)^49^ for assessing cognitive deficits in six tasks: verbal memory (BACS-VM), digit sequencing (BACS-DS), token motor task (BACS-TMT), semantic fluency (BACS-SF), symbol coding (BACS-SC), and tower of London (BACS-Tol). We computed the Z-scores of each BACS subtest and a composite Z-score of the total score of BACS^49^.

The participants of Study-A and Study-B were assessed for psychopathology symptoms on the 18-item Brief Psychiatric Rating Scale (BPRS) and the Positive and Negative Syndrome Scale (PANSS)^50,51^, respectively. In order to increase the sample size, we derived the BPRS scores from the PANSS item ratings in Study-B and analyzed them together with the BPRS scores in Study-A. We computed and analyzed the BPRS-total score, and four symptom domain weighted factor scores, including Affective (BPRS-AFF), Positive (BPRS-POS), Negative (BPRS-NEG), and Resistance (BPRS-RES)^52^.

### Group-level differences

The demographics and clinical characteristics of the participants are shown in Table 1. There were no significant differences in demographics among the three diagnostic groups, except that the duration of illness was significantly longer in SCZ (10.0, interquartile range (IQR): 5.0–17.0, years) than in MDD (3.5, IQR: 2.0–6.0, years). For overall negative symptoms indexed with NSA-Total, both SCZ (40.5, IQR: 36.0–47.0) and MDD (41.0, IQR: 35.0–45.0) had significantly more severe compared with HCs (29.0, IQR: 26.0–33.0), while no difference between SCZ and MDD. In terms of cognitive performance indexed with BACS-Composite Z-score, SCZ (−1.6, IQR: 36.0–47.0) had significantly worse performance than MDD (−0.16, IQR: −2.6 to −0.8), which in turn was significantly worse than HCs (0.0, IQR: −0.5 to 1.0). For general psychiatric symptoms assessed with BPRS-Total score, both SCZ (32.0, IQR: 27.0–38.5) and MDD (32.0, IQR: 29.0–37.0) had higher scores than HCs (20.0, IQR: 19.0–22.0). Although the BPRS-total score did not differ statistically between SCZ and MDD, MDD had more mood symptoms (BPRS-AFF, *p* < 0.005), and less positive (BPRS-POS, *p* < 0.005) and negative (BPRS-NEG, *p* < 0.05) than the SCZ group.

**Table 1:** Demographics, clinical information, and the number of digital records for all participants.

	MDD (*N* = 50)	SCZ (*N* = 103)	HCs (*N* = 75)	Tukey’s HSD test		
				P_SH_	P_DH_	P_DS_
Age (year)	32.5 (26.0, 49.0)	34.0 (27.0, 43.0)	34.0 (26.5, 43.0)	0.900	0.900	0.853
Gender (%)						
Male	26 (52.0)	50 (48.5)	38 (50.7)	0.900	0.900	0.900
Female	24 (48.0)	53 (51.5)	37 (49.3)			
Ethnicity (Chinese:Malay:India:Others)	36:5:6:3	87:7:9:0	54:16:4:1	0.514	0.427	0.059
Education years	14.5 (13.0, 16.0)	13.0 (11.5, 15.5)	14.0 (12.1, 15.5)	0.900	0.381	0.388
Duration of illness (years)	3.5 (2.0, 6.0)	10.0 (5.0, 17.0)	NA	NA	NA	<0.005
Medication						
CPZ equivalence (mg/day)	NA	333.3 (218.8, 729.2)	NA	NA	NA	NA
AntiDDosage (mg/day)	45.0 (25.0, 100.0)	NA	NA	NA	NA	NA
NSA-Total Score	40.5 (35.0, 45.0)	41.0 (36.0, 47.0)	29.0 (26.0, 33.0)	<0.005	<0.005	0.630
NSA-restricted speech	2.8 (1.9, 4.4)	2.8 (1.9, 3.8)	1.9 (1.9, 2.8)	<0.005	0.080	0.820
NSA-poor quality of speech	3.4 (1.6, 3.4)	3.4 (2.4, 4.3)	1.5 (1.5, 2.4)	<0.005	<0.005	0.186
NSA-affective blunting	6.1 (4.8, 7.9)	6.4 (3.9, 7.6)	3.6 (2.8, 4.7)	<0.005	<0.005	0.900
NSA-amotivation	9.9 (8.3, 11.0)	9.1 (7.9, 11.1)	5.6 (4.0, 6.6)	<0.005	<0.005	0.668
BACS-Composite Score	0.1 (−1.1, .8)	−1.6 (−2.6, −0.8)	0.0 (−.5, 1.0)	<0.005	<0.005	<0.05
BPRS-Total Score	32.0 (29.0, 37.0)	32.0 (27.0, 38.5)	20.0 (19.0, 22.0)	<0.005	<0.005	0.900
BPRS-affective	12.4 (10.4, 15.0)	8.1 (6.4, 9.4)	5.5 (4.5, 6.4)	<0.005	<0.005	<0.05
BPRS-positive	3.7 (3.7, 4.7)	7.3 (4.6, 9.5)	3.7 (3.7, 3.7)	<0.005	0.500	<0.005
BPRS-negative	7.0 (6.0, 8.9)	7.0 (5.2, 8.2)	4.7 (4.1, 5.1)	<0.005	<0.05	<0.05
BPRS-resistance	3.9 (3.1, 5.3)	4.6 (3.9, 5.9)	3.1 (3.1, 3.5)	<0.005	0.662	<0.005
Number of recordings						
Audio	48	98	70	NA	NA	NA
Video	42	44	45	NA	NA	NA
Kinect	42	92	66	NA	NA	NA
Audio or video	50	99	74	NA	NA	NA
Audio or kinect	50	103	75	NA	NA	NA
Video or kinect	42	92	66	NA	NA	NA
Audio or video or kinect	50	103	75	NA	NA	NA

Values are shown as median (IQR), unless otherwise indicated.

*MDD* major depressive disorder, *SCZ* schizophrenia, *HCs* healthy controls, *CPZ* chlorpromazine, *AntiDDosage* antidepressants medication dosage; *BACS* Brief Assessment of Cognition in Schizophrenia, *BPRS* Brief Psychiatric Rating Scale-18, *NSA* 16-item Negative Symptoms Assessment, *mg* milligram, *NA* not applicable.

### Prediction of negative symptoms

We utilized behavioral cues to infer the negative symptom severity in patients with SCZ and MDD. The prediction results of negative symptom total score (NSA-Total) in SCZ, MDD, and samples of all participant groups are presented in Table 2. The proposed method achieved a BAC (AUPRC) of 67.5% (0.673), 73.8% (0.778), and 75.3% (0.780) in differentiating between normal to mild and moderate to severe negative symptoms in SCZ, MDD, and all samples, respectively. In terms of predicting the factor scores of negative symptoms, as shown in Supplementary Table 2, our methods achieved better results for NSA-RS (BAC = 77.8%, AUPRC = 0.810) and NSA-AB (BAC = 78.0%, AUPRC = 0.837) than for NSA-PQ (BAC = 70.3%, AUPRC = 0.733) and NSA-AM (BAC = 67.7%, AUPRC = 0.709) across all samples. Additional results for predicting NSA-16 individual item scores, factor scores, and total scores with different combinations of behavior features are reported in Supplementary Table 3.

**Table 2:** Results for automated prediction of the severity of the negative symptoms, cognitive deficits, and psychopathology symptoms assessed by NSA-16, BACS, and BPRS.

Sample	Symptom domain	Scale	THR (severity)	Feature	SEN	SPE	PPV	NPV	AUPRC	BAC	MB
SCZ	Negative symptoms	NSA-Total	39.75 (mild/moderate)	F	0.850	0.500	0.607	0.786	0.673	67.5%	0.524
	Cognitive deficits	BASC-Composite	−1 (normal/mild)	VNF	0.667	0.788	0.611	0.825	0.726	72.7%	0.667
		BASC-Composite	−2 (mild/severe)	F	0.621	0.800	0.857	0.522	0.733	71.0%	0.659
	Psychopathology symptoms	BPRS-Total	32 (borderline/mild)	F	0.750	0.500	0.556	0.706	0.631	62.5%	0.545
MDD	Negative symptoms	NSA-Total	39.75 (mild/moderate)	F	0.850	0.625	0.654	0.833	0.778	73.8%	0.545
	Cognitive deficits	BASC-Composite	−1 (normal/mild)	VNFB	0.703	0.923	0.963	0.522	0.809	81.3%	0.740
	Psychopathology symptoms	BPRS-Total	32 (borderline/mild)	V	0.692	0.636	0.692	0.636	0.621	66.4%	0.542
SCZ+ MDD+ HCs	Negative symptoms	NSA-Total	39.75 (mild/moderate)	VNFB	0.711	0.796	0.835	0.655	0.780	75.3%	0.592
	Cognitive deficits	BASC-Composite	−1 (normal/mild)	VNFB	0.818	0.760	0.824	0.753	0.822	78.9%	0.579
		BASC-Composite	−2 (mild/severe)	VN	0.808	0.705	0.914	0.484	0.853	75.6%	0.796
	Psychopathology symptoms	BPRS-Total	24 (normal/ borderline)	N	0.688	0.813	0.874	0.581	0.758	75.1%	0.653
		BPRS-Total	32 (borderline/mild)	VN	0.833	0.638	0.565	0.871	0.772	73.6%	0.639

The scores are divided into binary classes by a clinically validated cutoff threshold (THR). Best prediction results for verbal (V), nonverbal (N), facial expression (F), and body movement (B) feature sets are presented. We computed the majority baseline (MB) of each prediction task as the performance benchmark, in which predictions default to the most frequent class.

*MDD* major depressive disorder, *SCZ s*chizophrenia, *HCs* healthy controls, *CM* confusion matrix, *SEN s*ensitivity, *SPE* specificity, *AUPRC* area under precision-recall curve, *PPV* positive predictive value, *NPV* negative predictive value, *BAC* balanced accuracy.

### Prediction of cognitive deficits

The prediction of BACS-composite scores and subscales are shown in Table 2 and Supplementary Table 2. For detecting mild to severe cognitive deficits (BACS-Composite < −1), the BACs (AUPRCs) for patients with SCZ, MDD, and all three types of subjects combined were 72.7% (0.726), 81.3% (0.809), and 78.9% (0.822), respectively. For detecting severe cognitive deficits (BACS-Composite < −2) in patients with SCZ and all three groups of subjects, the BAC (AUPRC) was 71.0% (0.733) and 75.6% (0.853), respectively. We did not detect severe cognitive performance in patients with MDD because only a small number of MDD patients (*N* = 4) have severe cognitive deficits in our dataset. For predicting BACS subscale scores (Above vs. Below, Supplementary Table 2), we achieved BACs above 70% for BACS-TMT in SCZ, BACS-VM, BACS-TMT, BACS-ToL, and BACS-SC in MDD, and BACS-SC in the mixed sample. More prediction results are listed in Supplementary Table 4.

### Prediction of general psychopathology

The prediction results of BPRS-Total and its 4-factor scores are shown in Table 2 and Supplementary Table 2. For general psychopathology indexed by BPRS-Total, only BACs larger than 70% emerged from the prediction of borderline (BPRS-Total ≥ 24) and beyond mild symptom severity (BPRS-Total ≥ 32) in the mixed sample, with a BAC (AUPRC) of 75.1% (0.758) and 73.6% (0.772), respectively. See Supplementary Table 5 and Supplementary Table 6 for additional prediction results using BPRS and PANSS.

### Classification of participants

In addition to predicting the severity of the symptoms, we also classified 50 patients with MDD, 103 patients with SCZ, and 75 HCs based on all the behavioral cues extracted from audio and video recordings (Table 3). Specifically, we performed a multi-category classification task on MDD vs. SCZ vs. HCs (BAC = 68.7%, AUPRC = 0.780) and pairwise classifications: MDD vs. SCZ (BAC = 84.7%, AUPRC = 0.905), MDD vs. HCs (BAC = 82.3%, AUPRC = 0.879), SCZ vs. HCs (BAC = 82.3%, AUPRC = 0.889), and patients vs. HCs (BAC = 79.8%, AUPRC = 0.863). Except for the multi-category classification task, the best classification results were obtained by fusing the prediction outputs from all feature sets (verbal, nonverbal, facial, and body movement). The detailed results for each type of feature set are shown in Supplementary Table 7.

**Table 3:** Results for automated classification of depression, schizophrenia, and healthy participants.

Task	Feature	SEN	SPE	PPV	NPV	AUPRC	BAC	MB
SCZ vs. HCs	VNFB	0.913	0.733	0.825	0.859	0.889	0.823	0.579
MDD vs. HCs	VNFB	0.740	0.907	0.841	0.840	0.879	0.823	0.600
MDD vs. SCZ	VNFB	0.874	0.820	0.909	0.759	0.905	0.847	0.673
MDD + SCZ vs. HCs	VNFB	0.773	0.822	0.682	0.880	0.861	0.798	0.670
MDD vs. SCZ vs. HCs	VNFB	0.680	0.840	0.778	0.761	0.780	0.687	0.452

The prediction results from the late fusion of verbal (V), nonverbal (N), facial expression (F), and body movement (B) are presented. We computed the majority baseline (MB) of each prediction task as the performance benchmark, in which predictions default to the most frequent class.

*SCZ* schizophrenia, *MDD* major depressive disorder, *HCs* healthy controls, *SEN* sensitivity, *SPE* specificity, *AUPRC* area under precision-recall curve, *PPV* positive predictive value, *NPV* negative predictive value, *BAC* balanced accuracy.

### System stability

To investigate the stability of behavioral cues and classification systems across two studies (Study-A and Study-B), we performed classification (SCZ vs. HCs) and negative symptoms severity prediction (borderline/mild vs. moderate/severe) on Study-A and Study-B separately and on individual sessions of Study-A, as shown in Table 5 and Table 4. In the first validation task, we trained the models on Study-A1 and B and tested them on Study-A2 and A3. For classifying patients with SCZ and HCs, the BAC (AUPRC) is 86.3% (0.950) and 83.0% (0.919) on Study-A2 and A3, respectively. For the prediction of NSA-Total on Study-A2 and A3, the BAC (AUPRC) ranges from 77.5% to 85.2% (0.832–0.865). In the second validation task, we trained models on data from Study-A1 and tested them on data of Study-B, and vice versa. When trained on Study-B and tested on Study-A1, the BACs (AUPRCs) for the classification and prediction tasks are 81.2% and 72.3% (0.844 and 0.781) for mixed samples, respectively, which shows that the models generalize well from Study-B to Study-A. However, when the models are trained on Study-A1 and tested on Study-B, the performance of the classification task is relatively poor (SCZ vs. HCs: BAC = 66.3%, AUPRC = 0.708). The drop in performance might be due to imbalance and the smaller number of participants in Study-A1 compared to Study-B.

**Table 4:** Results for classification of patients with schizophrenia and healthy controls across studies.

Task	Training session	Testing session	SEN	SPE	PPV	NPV	AUPRC	BAC	MB
SCZ vs. HCs	Study-A1 and Study-B	Study-A2	0.875	0.852	0.939	0.724	0.950	0.863	0.692
	Study-A1 and Study-B	Study-A3	0.864	0.796	0.929	0.655	0.919	0.830	0.690
	Study-A1	Study-B	0.694	0.735	0.706	0.723	0.750	0.714	0.500
	Study-B	Study-A1	0.692	0.889	0.857	0.750	0.834	0.791	0.675

The prediction results from *the* late fusion of verbal, nonverbal, and body movement are presented. We computed the majority baseline (MB) of each prediction task as the performance benchmark, in which predictions default to the most frequent class.

*SCZ* schizophrenia, *HCs* healthy controls, *SEN* sensitivity, *SPE* specificity, *F1* F1-score, *MCC* Matthews Correlation Coefficient, *AUPRC* area under precision-recall curve, *PPV* positive predictive value, *NPV* negative predictive value, *BAC* balanced accuracy.

**Table 5:** Results for predicting the severity of negative symptoms of patients with schizophrenia and depression and all participants across studies.

Samples	Scores	Training session	Testing session	THR	Feature	SEN	SPE	PPV	NPV	AUPRC	BAC	MB
MDD + SCZ	NSA-Total	Study-A1 and Study-B	Study-A2	39.75	N	0.708	0.905	0.895	0.731	0.845	0.807	0.533
		Study-A1 and Study-B	Study-A3	39.75	VN	0.846	0.857	0.880	0.818	0.865	0.852	0.553
		Study-A1	Study-B	39.75	N	0.712	0.614	0.685	0.643	0.708	0.663	0.542
		Study-B	Study-A1	39.75	N	0.862	0.762	0.833	0.800	0.844	0.812	0.580
MDD + SCZ + HCs	NSA-Total	Study-A1 and Study-B	Study-A2	39.75	VNB	0.760	0.860	0.760	0.860	0.832	0.810	0.632
		Study-A1 and Study-B	Study-A3	39.75	VNB	0.741	0.810	0.714	0.829	0.839	0.775	0.609
		Study-A1	Study-B	39.75	N	0.845	0.578	0.583	0.842	0.770	0.712	0.589
		Study-B	Study-A1	39.75	VNB	0.793	0.652	0.590	0.833	0.781	0.723	0.613

NSA-Total is divided into binary classes (above and below) by a cutoff threshold (THR). The THR of the NSA-Total score is set as the median on the training session. Best prediction results for verbal (V), nonverbal (N), and body movement (B) feature sets are presented. We computed the majority baseline (MB) of each prediction task as the performance benchmark, in which predictions default to the most frequent class.

*NSA* 16-item Negative Symptom Assessment, *SCZ* schizophrenia, *MDD* major depressive disorder, *HCs* healthy controls, *SEN* sensitivity, *SPE* specificity, *AUPRC* area under precision-recall curve, *PPV* positive predictive value, *NPV* negative predictive value, *BAC* balanced accuracy.

## Discussion

Inspired by earlier promising studies of digital phenotyping of psychiatric patients, we examined the relevance of a comprehensive portfolio of behavioral cues and signals extracted with state-of-the-art tools from the fields of signal processing and artificial intelligence for detecting psychiatric symptoms and discriminating between major diagnostic groups.

In this study, we examined the ability to detect a series of psychiatric manifestations, namely negative and general psychiatric symptoms, and cognitive performance. We summarized past studies that report classification and regression results of negative and general psychiatric symptoms using machine-learning techniques in Supplementary Table 8. For detecting negative symptoms, Cohen designed a model that, with 138 acoustic features as input, is able to predict blunted affect and alogia scores measured by the Scale for the Assessment of Negative Symptoms (SANS)^53^. Since their data is unbalanced, we calculated the BAC based on the metrics they provided for a fair comparison. Their results (blunted affect: BAC = 78.5%; and alogia: BAC = 81.0%) are almost in line with ours (NSA-AB: BAC = 78.0%; NSA-RS: BAC = 77.8%; Supplementary Table 2). We observed that the speech-related feature sets (verbal and nonverbal speech feature sets) are the most informative (Supplementary Table 4), which is consistent with earlier observations that vocal expressions are statistically significantly correlated with negative symptom measures, especially restricted speech and affective blunting^42,43^. We also found the prediction performance was better in the Diminished Expression (DE) domain indexed by NSA-RS (BAC = 77.8%, AUPRC = 0.810, Supplementary Table 2) and NSA-AB (BAC = 78.0%, AUPRC = 0.837, Supplementary Table 2) than Social Amotivation (SA) domain indexed by NSA-AM (BAC = 67.7%, AUPRC = 0.709, Supplementary Table 2). Furthermore, the prediction result of the DE and social SA domain score using PANSS (PANSS-DE, BAC = 83.5%; PANSS-AM, BAC = 65.7%; Supplementary Table 6) in SCZ further supports this observation.

To the best of our knowledge, we are the first to propose automated audio-visual-based methods for predicting the severity of cognitive deficits in SCZ or MDD. The proposed system can detect mild to severe cognitive deficits (BAC = 78.9%, AUPRC = 0.822, Table 2), as well as severe cognitive deficits (BAC = 75.6%, AUPRC = 0.853, Table 2) in a mixed sample consisting of all participants. For each patient group, our pipeline is able to detect cognitive deficits for MDD and SCZ with a BAC (AUPRC) of 81.3% (0.809) and 72.7% (0.726), respectively. In terms of the subscales of the cognitive battery, accurate predictions were consistently observed in BACS-TMT and BACS-SC across diagnoses. The BACS-TMT is a task measuring motor speed, and BACS-SC measures attention and speed of information processing, which are highly correlated with the expression domain of negative symptoms in SCZ^54^. Again, these results suggest that audio-visual behavioral characteristics are useful for predicting clinical ratings related to expression levels. In the long term, automated detection of cognitive symptoms may overcome some of the shortcomings of conventional assessments. For instance, BACS requires half an hour for a single standard battery of tests^49^, which could be avoided by automated prediction of BACS from short audio-visual recordings (e.g., phone calls).

For general psychiatric symptoms, we showcased that the proposed model is able to predict BPRS-Total in the mixed sample with robust results on the negative symptom factor score of BPRS but relatively poor results on the positive, effective, and resistance factor scores (Table 2 and Supplementary Table 2). A few studies in the literature found moderate to high correlations between machine-learning predictions and positive and negative symptoms indexed by BPRS and PANSS^22,55^. The results from these two studies, in combination with the present study, suggest that detecting general psychiatric symptoms from audio-visual behavioral cues is a promising avenue for future research. We observed that the prediction results of BPRS-Total in SCZ and MDD are relatively poor, but good results in the mixed sample, which may indicate that including samples with a broader distribution of general psychiatric symptoms benefits the differentiation of the symptom severity. Moreover, our model is able to differentiate BPRS-POS and BPRS-RES factor scales in MDD. Since most patients with MDD in our dataset did not present positive symptoms and resistance (Table 1), the prediction results indicated that there might have significant behavioral differences between symptomatic and asymptomatic patients with MDD.

In this study, we achieved a BAC of 84.5% in the classification of SCZ vs. MDD, 82.3% in the classification of SCZ vs. HCs, and 82.3% in the classification of MDD vs. HCs, when using verbal, nonverbal, and facial expressions, and body movement feature together. Past studies have reported an accuracy between 70% to 90% in the classification of SCZ and HCs, 70% and 95% in the classification of MDD and HCs, and 72.7% to 76.7% for differentiating MDD and SCZ (see Supplementary Table 9). Our results of distinguishing between patients and HCs are fair, while we achieved moderately high results on the classification task of MDD vs. SCZ. The existing studies that achieved a high accuracy (close to 90%) are often limited to a small number of patients^20,56^, did not perform cross-validation^57^ or strongly optimized the classifier at the risk of overfitting^24^. Therefore, those results might not be reliable. Furthermore, except for our previous preliminary studies^46^, all existing studies for SCZ assess a single type of behavioral cue (e.g., acoustic cues). In contrast, we recruited a larger number of patients with SCZ (*N* = 103), and integrated multiple types of behavioral cues compared to the existing literature. Overall, without optimizing the proposed pipelines to avoid overfitting, we still achieved good accuracy, which supports the effectiveness of audio-visual features for distinguishing the diagnostic groups.

In system stability analysis, we observe that the proposed system performs consistently across different time points (the last two sessions from Study-A) and for the two independent cohorts (Study-A vs. Study-B) for recognizing SCZ from HCs and predicting the severity of negative symptoms. The results suggest that the classification and prediction models might generalize to other recording conditions as long they are trained on sufficiently large datasets. As far as we know, such out-of-distribution tests on independent datasets have not been conducted before in this context, yet those tests are crucial for assessing how robustly the machine-learning pipelines can handle varying recording settings, different recording devices, different patients, different demographics, etc. We believe this might be the first study to investigate how well a machine-learning pipeline for digital behavioral phenotyping generalizes across different time points and different studies. These results seem to support our long-term goal of designing low-cost recording technologies for the continuous monitoring of patients.

In summary, the findings in the present study demonstrate that important and relevant clinical features in major psychiatry disorders can be detected from audio-visual behavioral data by machine-learning methods. Although the results are promising, independent replication and further technology development is required for this machine-learning technology to realize its full potential for accurate and unbiased remote long-term psychiatric assessment. Implemented as a smartphone app or a virtual healthcare application, such a pipeline may provide valuable early diagnosis and longitudinal monitoring of severe mental illnesses. In the future, we hope to expand the studies at multiple institutions to cover participants with a wide variety of cultural and ethnic backgrounds. We also plan to apply the proposed pipelines to phone calls, which may expand the reach and impact of the technology.

### Limitations

This study has the following limitations. Most of the patients involved in this study exhibit mild to moderate symptoms. It is vital to develop multi-center datasets to enlarge the sample size and balance the spectrum distribution of symptom severity. For this complex modeling, the group of patients is still small. Moreover, since all participants in both studies were of Asian ethnicity, and behavioral patterns might differ between cultures and ethnic groups, it is necessary to validate our models in populations with diverse ethnicities and cultures in future studies. Furthermore, the automatic speech recognition and facial analysis tools deployed in this study were trained on data collected in the United States; hence they may perform less reliably on the data of the present study^58^. Finally, the data for the present study were collected during three visits over a period of only 12 weeks. Long-term data collection of a larger group of patients will be required in the future.

## Methods

In this study, we collected audio, video, and Kinect recordings from conversations in NSA-16 interviews with 228 participants (103 SCZ, 50 MDD, and 75HC). The diagram of the analysis pipeline is shown in Fig. 1. We applied the speaker diarization technique to recognize the participant’s speech and the speech recognition toolkit to transcribe the participant’s speech to text. Then, we extracted four verbal feature sets from the transcriptions to measure the linguistic characteristics (e.g., word frequency) and three nonverbal feature sets from the participant’s raw speech data to measure acoustic, prosodic, and conversational features (e.g., pitch, intensity, and response time). From the video recording, we leverage three facial expression feature sets to measure facial emotion, movement of facial landmarks, head movement, and eye gaze. The three-dimensional body movement features were extracted from the Kinect recordings. Finally, we trained ensemble machine-learning algorithms on those feature sets to classify participant groups and predict the clinical assessment ratings.

In the following, we first explain the participant and experimental procedure. Second, we elaborate on how we extract 11 behavioral feature sets from the audio, video, and Kinect recordings of the interviews. At last, we discuss how we binarize the clinical assessment scores and explain the proposed ensemble learning model that integrates those numerous features.

### Participant

**Fig. 2:**
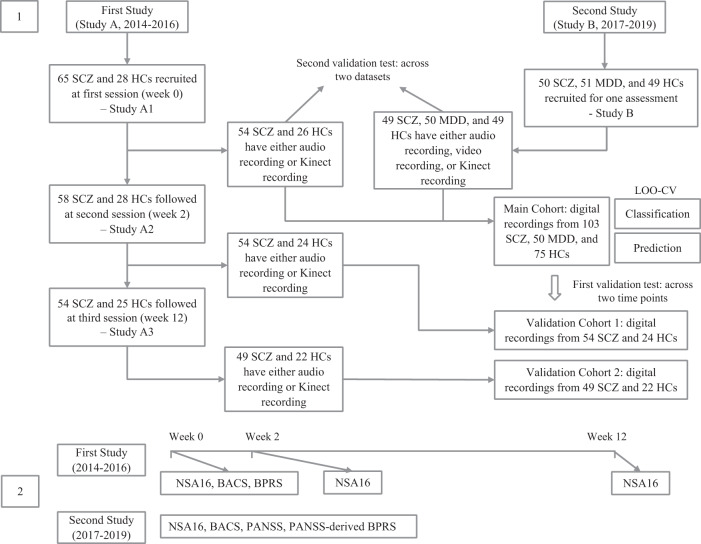
Patients flow diagram and assessment scales. Study A was a longitudinal study with three study visits including 65 SCZ and 28 HC, and Study B was a cross-sectional study including 50 SCZ, 51 MDD, and 49 HC. The data collected from Study A baseline visit and Study B were combined to validate the machine learning performance. The data collect in each session were cross validated in order to investigate the stability of behavioral cues and classification systems.

We analyzed the data from two studies (see Fig. 2). Study-A was designed to elucidate objective features extracted from audio and video recordings, for assessing social behavior in patients with schizophrenia and explore the ability of those features in prognosticating the outcome of cognitive remediation therapy (CRT). Participants were assessed at baseline, 2-week, and 12-week time point. Study-B was designed to explore the specific speech and motor cues for mapping against the severity of negative symptoms, neurocognitive impairments, and social-cognitive deficits in schizophrenia and depression. All participants with SCZ in both studies were recruited from the outpatient clinics at the Institute of Mental Health (IMH), Singapore, and HCs from the general population. For participants with MDD in Study-B, 19 (38%) participants were recruited from the inpatient ward, and the rest were recruited from the outpatient clinics in IMH. The inclusion criteria in both studies were aged 21–65 years, English-speaking, having the capacity to provide informed consent, diagnosis of SCZ or MDD for the patient group, and no history of any mental disorder for HCs. The exclusion criteria for participants in both studies included a history of strokes, traumatic brain injuries, and neurological disorders. The diagnoses of SCZ and MDD were ascertained on the Structured Clinical Interview for DSM-IV (SCID-I/P), and HCs were screened using the non-patient version (SCID-I/NP)^59^. Both studies were approved by the National Healthcare Group’s Domain Specific Review Board, Singapore. All participants provided written informed consent. There was no overlap between the samples of the two studies.

The descriptive analysis of SCZ, MDD, and HCs in two studies and their comparisons are presented in Supplementary Table 1. The main difference is that the age of the participants in Study-B is greater than that of Study-A, and participants with SCZ in Study-B have a longer duration of illness than participants with SCZ in Study-A. Because there was no difference in age, gender, education, and ethnicity between the SCZ group and HC group in both studies, the data collected from Study-A baseline visit and Study-B were combined to train machine-learning algorithms.

### Experimental procedure

During our experiments, all participants were evaluated at the Institute of Mental Health (IMH) in Singapore. Specifically, audio, video, and Kinect skeleton data were recorded during semi-structured interviews of NSA-16 in both studies, lasting 28 min on average. The illustration of the data collection interview is shown in Fig. 1. The voice of both the participant and the psychiatrist were recorded through two separate lapel microphones. These two microphones were connected to an H4N recorder which captures the two-channel speech signals at 48 kHz. We recorded RGB and depth data through Microsoft Kinect for both studies. The participants were seated in a fixed position. A webcam was pointed at the face of the participants and recorded video at 1080p resolution with a frame rate of 15 fps (only for the second study). The psychiatrist and the Kinect device are approximately 2.5 meters away from the participant, while the webcam is about 1 meter away from where the participant is sitting. All the digital recordings were recorded and stored on a laptop, and several backups were made on external hard drives.

### Behavioral features extraction

#### Data preprocessing

Before analyzing the recordings, we conducted two preprocessing steps. We first manually removed the segments recorded during the installation and removal of the recording equipment. Second, in order to reduce the impact of environmental noise and the varying distance from the microphone to the participant on the recording quality, we applied the Audacity software (https://www.audacityteam.org/) and the FFmpeg-normalized toolkit (https://github.com/slhck/ffmpeg-normalize) to reduce the noise and normalize the volume, respectively; typically, we achieved a noise reduction of 6 dB and a mean volume level of −26 dB. The noise statistics were automatically extracted from manually selected noisy segments.

#### Speaker diarization

In this study, we do not analyze the speech of the psychiatrist. Instead, we only assess the speech of the participants. To automatically extract the speech of the participants in the recordings, we apply speaker diarization techniques. We implemented a Hidden Markov Model (HMM) to extract binary sequences from both audio channels to identify *who* is speaking and when^60^. The binary sequence contains zeros whenever the participant is not speaking and ones otherwise. In addition, to obtain cohesive speech segments for speech recognition, we apply a one-dimensional closing operation (erosion of the dilation) to the binary sequences^61^. Finally, by multiplying the participant’s audio channel with the corresponding binary mask, we obtain a speech signal with only the participant’s voice.

#### Speech recognition

After extracting the participant’s speech, we applied the Kaldi speech recognition toolkit for automatically transcribing the participant’s speech into text files. More specifically, we utilize the pre-trained ASpIRE Chain model for automated transcription^62^. This model is a DNN-HMM model, combining a deep neural network (DNN) with a HMM, pre-trained on Fisher English recordings^63^, augmented with impulse responses and noises to create a multi-condition training dataset.

#### Verbal features

We extracted verbal features through the bag-of-words models LIWC 2015 and Diction 7.0 software^64,65^, which both extract the occurrence of words within a document. The LIWC features comprise the word counts for 77 categories, including 21 linguistic counts (function words, common verbs, adjectives, etc.), 40 categories related to psychological processes (words related to affect, sociality, cognition, perception, drive, etc.), 6 informal language markers (assents, fillers, swear words, question marks, netspeak, and informal words), 7 personal concern categories (work, home, leisure activities, etc.), and 3 general text metrics (the number of unique words, words in LIWC dictionary, and words with more than six letters). Similarly, Diction 7.0 generates 5 master features (Activity, Optimism, Certainty, Realism, and Commonality), 35 sub-features (e.g., Denial, Accomplishment, Present Concern, Centrality, Insistence, etc.), and 2 text metrics (number of unique words and average word size). Specifically, the 5 master features were composed of several standardized sub-features via addition and subtraction. We normalized the LIWC category counts and Diction sub-features by the total number of words.

Apart from word-based tools, we also converted transcriptions into a vector space employing two unsupervised models: latent Dirichlet allocation (LDA) and Doc2Vec^66,67^. The LDA is a statistical model used to identify different topics of documents. Each document is modeled as a multinomial distribution of topics, and each topic is modeled as a multinomial distribution of words. It automatically generates the categories and their associated word probabilities, hence there is no need to craft them manually. We first generated the top 100 topics by LDA from transcripts of the speech of the participants, where we trained LDA models on the text files in training set in each cross-validation (CV) loop with predefined epoch and random seed, and we obtained the LDA topics from the text files in the test set by applying the trained models. Next, for each topic, we selected the top 50 words with the largest word probability and counted the number of these words that appeared for each document. Finally, we normalized these counts by the total number of words. The resulting normalized counts were treated as features for classification and prediction.

Moreover, we leveraged the Doc2Vec model to generate a document vector from each transcription in each CV loop. Specifically, we create document vectors using the Distributed Memory of Paragraph Vector (PV-DM) algorithm^67^ implemented in the Gensim library^68^. The PV-DM algorithm lets the model randomly sample consecutive words from a paragraph and predicts a center word from the randomly sampled set of words. We set the length of the document vector to 100. The document vectors are regarded as features for prediction and classification tasks.

#### Non-verbal features

Besides analyzing the linguistic content of the speech of the participant, we also computed low-level acoustic and prosodic descriptors by applying the OpenSMILE and DisVoice toolkits^69,70^. The low-level descriptors (LLDs) extracted by OpenSMILE and DisVoice toolkits are summarized in Supplementary Table 10. The OpenSMILE toolkit is a modular and adjustable collection of acoustic features useful for signal processing and machine-learning applications. Specifically, we employed the ‘emobase_live4’ configuration of OpenSMILE to extract the following LLDs: intensity, loudness, 12 MFCCs, pitch (F0), probability of voicing (VoiceProb), F0 envelope (F0env), 8 line spectral frequencies (LSF), and Zero-Crossing Rate (ZCR). Moreover, the following functions are applied to the LLDs and their delta coefficients: minimum and maximum values and their relative position from input (minPos and maxPos), range, mean, 2 linear regression coefficients (linregc1–2), linear and quadratic error, standard deviation (STD), skewness, kurtosis, values in 3 quartiles (quartile1–3), and 3 interquartile ranges (e.g., iqr1-2, iqr2-3, and iqr1–3). These LLDs and functions represent a speech utterance with 988 features. Before computing the LLDs, we first removed pauses and silences from the participant’s speech, resulting in a continuous speech signal without silences. Then we extracted 988 emotion-based prosodic features from the entire speech with a 100 ms sliding window. Finally, the maximum, minimum, mean, and standard deviation of these emotion-based features were composited as the openSMILE features (a total of 3952 features).

We also applied the DisVoice toolkit to the speech signals, which was first developed specifically for quantifying speech deficits of patients with Parkinson’s disease^70^. The DisVoice toolkit provides articulation, prosody, and phonation features. The articulation features include the mean, STD, skewness, and kurtosis of the following speech measures: first formant frequency (FF1), second formant frequency (FF2), 22 bark band energies (BBEs), and 12 MFCCs with both onset (from unvoiced to voiced) and offset (from voiced to unvoiced) transitions, where we also measured the first and second derivative of these features (e.g., DMFCC and DDMFCC). The prosody features include duration-based, F0-based, and energy-based measures. In the following, we briefly describe those three types of components. The duration-based features comprised the mean, STD, minimum, and maximum duration of the voiced segments and pauses (VoiceDur and PauseDur); the number of voiced segments and pauses per second (VoicedRate and PauseRate); and voiced and unvoiced duration regularity (VDR and UDR). The F0-based features consist of the average tilt and tilt regularity of F0 (F0_slope_mean and F0_slope_std), linear regression coefficients extracted from the F0 contour (F0_regcoef), and the mean, STD, and maximum F0 in voiced segments in both hertz and semitones (F0_Hz and F0_semitones). The energy-based features comprise the voiced/unvoiced energy regularity, the average tile of the energy contour (EnergySlope_mean), the linear regression coefficients extracted from the energy contour (Energy_regcoef), mean square error of the reconstructed energy contour with a 1-degree polynomial (Energy_mae), mean and STD of delta energy within consecutive voiced segments, and mean, STD, and maximum of logarithmic energy (LogE). At last, phonation features were computed over the voiced segments, including the mean, STD, skewness, and kurtosis of the following measures: jitter, shimmer, amplitude perturbation quotient (APQ), pitch perturbation quotient (PPQ), LogE, and the first and second derivative of F0 (DF0 and DDF0).

Moreover, we assessed the interactions between participants and psychiatrists in this study, similarly to our early research^20^. We calculated 14 conversational features from the speech of the participant and psychiatrist, extracted by speaker diarization: the number of short utterances per minute (Interject), the number of interruptions per minute (Interrupt), the average response time of the participant (Response Time), average turn duration (Turn Duration), the percentage of speech (Speaking), the average duration of silence/pause (Speech Gap), the difference in the speaking percentages (Difference Speaking), the difference of natural turns (Difference Turn), word count per second (Speaking Rate), percentage of no speaking (Mutual Silence), percentage of duration when both speakers are speaking (Overlap), number of failed interrupts (Failed Interrupt), number of short utterances when another speaker is speaking (Speaking Interject), and the number of turns without interruption (Natural Turn). Some of those conversational cues are illustrated in Supplementary Figure 1.

#### Facial expression features

Besides speech signals, we also examine the affective expression on the face of the participants. We applied three different toolkits to compute facial features: Affectiva^71^, OpenFace^72^, and Opsis^73,74^. In each case, we processed the entire video recordings of the interviews. In other words, we did not select specific episodes or events during the clinical interviews but analyzed the full videos instead. In the following, we summarize the facial expression cues considered in this study.

The Affectiva toolkit calculates the probability value of 7 emotions (Anger, Contempt, Disgust, Fear, Joy, Sadness, and Surprise), 2 composite emotional metrics (Engagement and Valence), 20 facial motions (e.g., MouthOpen, CheekRaise, NoseWrinkle, ChinRaise, EyeClosure, LipStretch, Smirk, etc.), and 13 emojis (e.g., Laughing, Smiley, Wink, Relaxed, Scream, StuckOutTongue, etc.). In addition, the Opsis toolkit (http://www.opsis.sg/) quantifies facial expressions in a three-dimensional continuous space: Arousal (passive vs. energetic), Valence (negative vs. positive), and Intensity (difference from neutral). Besides these three emotional metrics, 3 head postures (Roll, Pitch, and Yaw angles) and 1 eye openness feature (Lambda) are also measured by the Opsis toolkit^73,74^. At last, we also applied the OpenFace toolkit^72^ to quantify facial expressions. This toolkit automatically captures 2 eye-gaze directions in world coordinates (GazeAngle x for vertical axis and GazeAngle y for the horizontal axis); 6 rigid shape parameters including scale (P_scale), rotation (P_rx, P_ry, and P_rz), and translation (P_tx and P_ty) terms; 34 non-rigid shape parameters (NSP0 to NSP33); the regression intensity of 17 Facial Action Units (AU01_reg to AU17_reg); and the classification values of these AUs in a binary format (AU01_clf to AU17_clf). Facial expressions captured by Affectiva and OpenFace were illustrated in Supplementary Figure 2.

We calculated the differences in the features across consecutive frames (referred to as delta values), indicating how much the features change over time. Next, we computed statistical measures of those features across the entire length of the videos. Specifically, we calculated the mean, minimum, maximum, median, skewness, and kurtosis of all Affectiva and Opsis features (except the three head postures) and their delta values. In addition, we also included the percentage of Affectiva scores above a threshold of 10 (maximum is 100) into the Affectiva feature set to measure the duration of emotions and facial expressions, where a threshold of 10 can capture most expressions without being affected by noise. Finally, for OpenFace features extracted across consecutive frames, we calculated the mean of AUs classification values and the mean, minimum, maximum, median, skew, and kurtosis values of other OpenFace features (face shape parameters and gaze direction).

#### Body movement features

We automatically extracted skeletal points from the Microsoft Kinect depth recordings. The names of those joints are shown in Supplementary Figure 3. We first applied a median filter with a one-second sliding window to remove spike noise. Next, we measured the linear speed (LinSpeed) of all 20 joints by calculating the differences between adjacent frames, and we computed the mean and STD of the linear speed values. Apart from the linear velocity of the joints, we also evaluated the angular speed (AngSpeed) and acceleration (AngAcc) of 6 body angles (left and right shoulder, elbow, and wrist joints). Similarly, we also calculated the mean and STD for all angular speeds and accelerations. The resulting 64 features constitute the body movement feature set.

### Label binarization

Most of the patients in our study have only mild symptoms. Therefore, the clinical assessment scores do not cover the entire range but typically take low values. To predict the clinical assessment scores, we divided those scores into two classes, distinguishing the severity of symptoms on two levels only. In other words, each subjective rating was split into class *Below* (score < threshold) and class Above (score ≥ threshold).

Following the mapping between NSA-16 and Clinical Global Impression-Schizophrenia scale (CGI-SCH), we determined the cutoff score of NSA-Total for mild and moderate severity to be 39.75, which is the mean value between the mildly ill and moderately ill^75^. For BPRS and PANSS ratings, we set the cutoff scores of PANSS-FSNS, PANSS-Total, and BPRS-Total by means of the equipercentile linking results on the Clinical Global Impressions-Severity (CGI-S) scale^76^, since these thresholds have more clinical significance^77–79^. The equipercentile linking approach maps those scores to CGI-S with the same percentile ranks. Following this approach, the cutoff scores of PANSS-Total and PANSS-FSNS between normal and borderline severity are set to 38 and 9.5, respectively; the cutoff score of PANSS-Total and PANSS-FSNS for borderline and mild severity is set to 52 and 14.5, respectively; the cutoff scores of BPRS-Total between normal and borderline and between borderline and mild severity are set to 24 and 32, respectively. For cognitive symptoms, we consider the thresholds −1 and −2 on the BACS-composite values, representing one and two standard deviations below the scores of healthy subjects; by means of those thresholds, we define normal (score > −1), mild (−1<score < −2), and severe (score < −2) cognitive symptoms according to the BACS-composite scale^80^. For factor scales and subscales used in this study, there are no rigorous clinically relevant cutoff scores as far as we know. Therefore, we select the median values or values close to the median values on the training data as the cutoff score such that the counts of both classes are as similar as possible. In this manner, the data is well-balanced between the two categories (*Above* and *Below*).

### Classification method

**Fig. 3:**
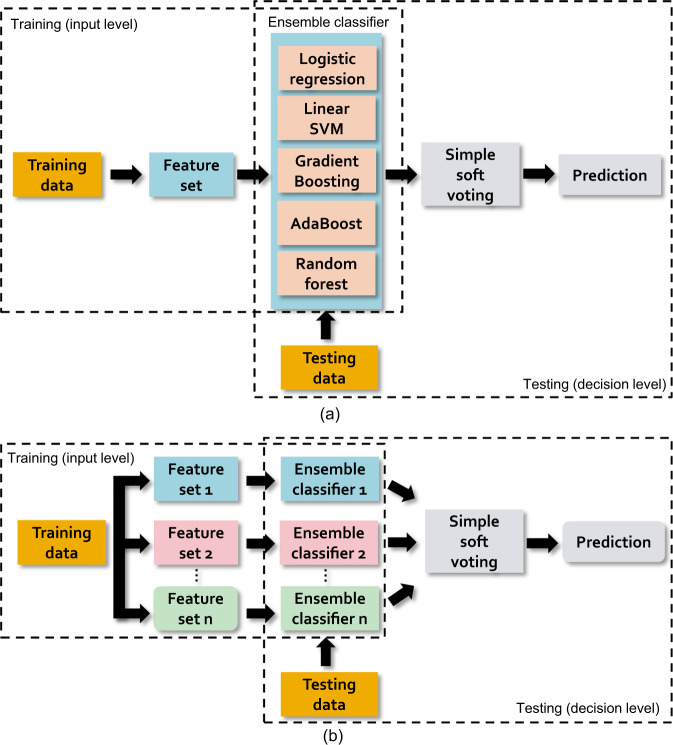
The pipeline of ensemble learning. The diagram in **a** shows how we classify each feature set individually, while the diagram in **b** illustrates how we combine votes from multiple feature sets by late fusion to generate the final prediction.

As explained in the previous sections, we extracted 11 different feature sets from the interview recordings: 4 verbal feature sets (LIWC, Diction, LDA, and Doc2Vec), 3 nonverbal speech feature sets (Conversational, OpenSMILE, and DisVoice), 3 facial feature sets (Affectiva, OpenFace, and Opsis), and one body movement feature set. Subsequently, we proposed an ensemble learning pipeline (see Fig. 3) to predict subjective assessment scores (prediction tasks) from those numerous features and to classify the different participant groups (classification tasks). We formed the ensemble learning pipeline and implemented all classification and prediction tasks based on the Scikit-learn toolkit (version 0.23.2) in Python 3.8.

We validate all classification and prediction tasks through leave-one-out cross-validation (LOO-CV). In LOO-CV, we train the models on N-1 subjects, test the model on the data of the left-out subject, and repeat this procedure for all N subjects. The performance of the models is averaged across all N left-out participants. We train a separate ensemble classifier for each feature set separately. Each of those ensemble classifiers contains five base classifiers: Support Vector Machine with linear kernels, Logistic Regression, Gradient Boosting, AdaBoost, and Random Forest. We fixed the hyperparameters and the random seeds for those five base classifiers in order to generate reproducible results. The hyperparameters are listed in Supplementary Table 10. To create a robust classification pipeline, we combined five common classifiers instead of relying on only a single classifier, did not optimize the parameters of the classifiers but chose the standard settings instead, and integrated multiple types of behavioral cues. The proposed pipeline generated numerous “votes” from each component classifier and for each kind of behavioral signal. Next, the system made a decision (e.g., “SCZ” or “HCs”) based on majority voting. Notably, when we validate our results across Study-A, we applied leave-one-subject-out cross-validation to provide a rigorous evaluation: all data from the same subject is either in the training or test set; therefore, data from the same subject is never included in both the training and test set.

Before combining the outputs of the base classifiers, we standardized those predictions from each feature set in a non-trivial manner (referred to as probability calibration). We first applied an internal LOO-CV to obtain the probability outputs on the training set. Next, the minimum, maximum, and optimal threshold of these probability outputs on the training set were used to calibrate the predictions of the test set into a range of 0 and 1 for each of the five base classifiers, where the optimal threshold is determined as the decision threshold with the maximum geometric mean score. At last, the standardized predictions from all feature sets are combined by averaging, resulting in the final prediction based on all feature sets.

In addition, we applied the Synthetic Minority Oversampling Technique (SMOTE)^81^ to overcome the class imbalance, which creates synthetic data for the minority class by interpolating existing data points. We also applied z-score standardization to all features, where we subtracted the mean from each feature value and divided it by the standard deviation. As a result, the standardized features have a mean of 0 and a standard deviation of 1.

To evaluate the classification and prediction performance, we calculate and present several standard classification metrics: sensitivity (SEN), specificity (SPE), positive predictive value (PPV), negative predictive value (NPV), balanced accuracy (BAC), and area under the precision-recall curve (AUPRC). We mainly discuss the classification and prediction results based on the BAC, since it is a good metric to deal with imbalanced data. It is the arithmetic mean of SEN and SPE. We also briefly discussed the AUPRC for the classification and prediction tasks, which is also a valuable metric for imbalanced machine-learning problems.

## Supplementary information

Revised online Supplementary file

## Data Availability

The datasets analyzed during the current study are not publicly available due to participant privacy and security concerns. The dataset is stored inside the IMH and cannot be accessed outside. Qualified researchers may contact the corresponding author for more information.

## Code availability

Due to the agreement with the hospital, we cannot share the code and data publicly. Please contact the corresponding author to request the code used for data processing and analysis.
