# Supplementary material for: Identifying psychiatric manifestations in schizophrenia and depression from audio-visual behavioural indicators through a machine-learning approach
Source: Schizophrenia (Heidelb). 2022 Nov 7;8(1):92. doi: 10.1038/s41537-022-00287-z (PMC9640655; doi:10.1038/s41537-022-00287-z)
Supplement: Supplementary file 1 — Revised online Supplementary file [file 41537_2022_287_MOESM1_ESM.pdf]

Supplementary Information for

Identifying Psychiatric Manifestations in Schizophrenia and Depression from Audio-Visual Behavioural Indicators through a Machine-Learning Approach

Shihao Xu[1+], Zixu Yang[2+], Debsubhra Chakraborty[1], Yi Han Victoria Chua[1], Tolomeo Serenella[3], Stefan Winkler[4], Michel Birnbaum[5], Bhing-Leet Tan[2], Jimmy Lee Chee Keong[2,6], and Justin Dauwels[7*]

[1] School of Electrical and Electronic Engineering, Nanyang Technological University, Singapore; [2] Institute of Mental Health, Singapore; [3] Department of Psychology, National University of Singapore, Singapore; [4] School of Computing, National University of Singapore, Singapore; [5] Mindsigns Health, Singapore; [6] Lee Kong Chian School of Medicine, Nanyang Technological University, Singapore; [7] Faculty of Electrical Engineering, Mathematics, and Computer Science, Delft University of Technology, Delft, Netherlands

* Corresponding author: Justin Dauwels (j.h.g.dauwels@tudelft.nl)

Supplementary Table 1: Descriptive analysis of participant groups in the first and second study.

	Study A		Study B						
	Schizophrenia (N=54)	Controls (N=26)	Depression (N=50)	Schizophrenia (N=49)	Controls (N=49)	P_{SH1}	P_{SH2}	P_{SS}	P_{HH}
**Age (years)**	29.0 (25.0, 37.0)	28.0 (22.2, 34.5)	32.5 (26.0, 49.0)	41.0 (32.0, 47.0)	38.0 (30.0, 49.0)	0.377	0.812	<0.005	<0.005
**Gender (%)**									
**Male**	25 (46.3)	12 (46.2)	26 (52.0)	24 (49.0)	23 (46.9)	0.991	0.842	0.788	0.949
**Female**	29 (53.7)	14 (53.8)	24 (48.0)	25 (51.0)	26 (53.1)				
**Education years**	13.4 (12.0, 14.9)	13.5 (12.4, 15.0)	14.5 (13.0, 16.0)	13.0 (11.5, 15.5)	14.0 (11.0, 16.0)	0.849	0.948	0.794	0.985
**Ethnicity(Chinese:Malay:India:Others)**	47:5:3:0	22:3:1:0	36:5:6:3	41:2:6:0	32:13:3:1	0.927	0.247	0.492	0.104
**Duration of illness (years)**	6.5 (3.2, 13.5)	NA	3.5 (2.0, 6.0)	13.0 (7.0, 20.0)	NA	NA	NA	<0.005	NA
**Medication**									
**CPZ equivalence (mg/day)**	312.5 (166.7, 687.5)	NA	NA	427.1 (250.0, 763.0)	NA	NA	NA	0.139	NA
**AntiDDosage (mg/day)**	NA	NA	45.0 (25.0, 100.0)	NA	NA	NA	NA	0.139	NA
**NSA Total Score**	40.5 (36.0, 47.0)	26.5 (24.0, 29.8)	40.5 (35.0, 45.0)	41.0 (36.0, 48.0)	30.0 (27.0, 37.0)	<0.005	<0.005	0.860	<0.005
**NSA-Restricted Speech**	3.2 (1.9, 3.8)	1.9 (1.9, 2.6)	2.8 (1.9, 4.4)	2.8 (1.9, 3.8)	1.9 (1.9, 3.7)	<0.005	0.138	0.917	<0.05
**NSA-Poor Quality of Speech**	3.4 (1.5, 4.3)	1.5 (1.5, 1.5)	3.4 (1.6, 3.4)	3.4 (2.4, 3.9)	2.4 (1.5, 2.5)	<0.005	<0.05	0.219	<0.005
**NSA-Affective Blunting**	5.7 (3.8, 7.5)	3.5 (2.8, 3.7)	6.1 (4.8, 7.9)	6.8 (4.1, 8.5)	3.7 (2.8, 5.3)	<0.005	<0.005	0.134	<0.05
**NSA-Amotivation**	9.2 (7.5, 11.3)	5.1 (4.0, 6.3)	9.9 (8.3, 11.0)	9.1 (7.9, 10.6)	6.0 (4.1, 6.8)	<0.005	<0.005	0.617	0.120
**BACS Composite Score**	-1.7 (-2.7, -0.8)	0.5 (-0.2, 1.1)	0.1 (-1.1, 0.8)	-1.5 (-2.5, -0.9)	-0.1 (-0.8, 0.5)	<0.005	<0.005	0.230	<0.05
**BPRS Total Score**	32.0 (24.5, 39.5)	19.5 (18.0, 21.0)	32.0 (29.0, 37.0)	31.0 (28.0, 37.0)	20.0 (19.0, 22.0)	<0.005	<0.005	0.904	0.070
**BPRS-Affective**	8.1 (6.5, 9.4)	4.5 (4.5, 5.5)	12.4 (10.4, 15.0)	8.4 (6.4, 9.4)	5.5 (4.5, 6.5)	<0.005	<0.005	0.271	0.087
**BPRS-Positive**	7.4 (4.6, 9.5)	3.7 (3.7, 3.7)	3.7 (3.7, 4.7)	6.7 (4.7, 9.5)	3.7 (3.7, 3.7)	<0.005	<0.005	0.747	0.203
**BPRS-Negative**	6.9 (5.1, 7.9)	4.1 (4.1, 4.7)	7.0 (6.0, 8.9)	8.0 (6.0, 9.9)	5.1 (4.1, 5.1)	<0.005	<0.005	<0.05	<0.05
**BPRS-Resistance**	4.6 (3.1, 6.9)	3.1 (3.1, 4.0)	3.9 (3.1, 5.3)	4.6 (3.9, 5.4)	3.1 (3.1, 3.1)	<0.005	<0.005	0.170	<0.05
**Number of Recordings**									
**Audio**	50	25	48	48	45	NA	NA	NA	NA
**Video**	NA	NA	42	44	45	NA	NA	NA	NA
**Kinect**	47	21	42	45	45	NA	NA	NA	NA
**Audio or Video**	50	25	50	49	49	NA	NA	NA	NA
**Audio or Kinect**	54	26	50	49	49	NA	NA	NA	NA
**Video or Kinect**	47	21	42	45	45	NA	NA	NA	NA
**Audio or Video or Kinect**	54	26	50	49	49	NA	NA	NA	NA

Values are shown as median (IQR), unless otherwise indicated. The group demographics and assessment scores are compared by t-tests. Abbreviation: P_{SH1}=p-value between schizophrenia and control groups of the first study; P_{SH2}=p-value between schizophrenia and control groups of the second study; P_{SS}=p-value between schizophrenia groups of two studies; P_{HH}=p-value between health control groups of two studies; CPZ=Chlorpromazine; BACS=Brief Assessment of Cognition in Schizophrenia; BPRS=Brief Psychiatric Rating Scale-18; NSA=16-item Negative Symptoms Assessment; mg=milligram; NA=Not Applicable.

Supplementary Table 2: Results for automated prediction of the subscales.

Sample	Symptom domain	Scale	THR	Feature	SEN	SPE	PPV	NPV	AUPRC	BAC	MB
S	Negative symptoms	NSA-RS: Restricted speech	3.637	N	0.822	0.774	0.755	0.837	0.818	79.8%	0.541
		NSA-PQ: Poor Quality of Speech	3.359	VN	0.789	0.488	0.682	0.625	0.597	63.9%	0.582
		NSA-AB: Affective Blunting	6.371	VNFB	0.712	0.725	0.725	0.712	0.713	71.9%	0.505
		NSA-AM: Amotivation	9.162	F	0.571	0.826	0.750	0.679	0.718	69.9%	0.523
	Cognitive deficits	BASC-VM: Verbal memory	-0.790	VNFB	0.600	0.566	0.566	0.600	0.568	58.3%	0.515
		BASC-DS: Digit sequencing	-0.800	B	0.600	0.553	0.563	0.591	0.568	57.7%	0.511
		BASC-TMT: Token motor task	-1.260	VN	0.647	0.872	0.846	0.695	0.739	76.0%	0.520
		BASC-SF: Semantic fluency	-1.010	N	0.653	0.571	0.604	0.622	0.595	61.2%	0.500
		BASC-SC: Symbol coding	-1.520	N	0.580	0.625	0.617	0.588	0.533	60.3%	0.510
		BASC-ToL: Tower of London	-0.060	VN	0.404	0.765	0.613	0.582	0.510	58.4%	0.520
	Psychopathology symptoms	BPRS-AFF: Affective	8.390	V	0.592	0.735	0.690	0.643	0.627	66.3%	0.500
		BPRS-POS: Positive	7.300	V	0.680	0.646	0.667	0.660	0.630	66.3%	0.510
		BPRS-NEG: Negative	7.020	N	0.809	0.588	0.644	0.769	0.701	69.8%	0.520
		BPRS-RES: Resistance	4.740	F	0.650	0.667	0.619	0.696	0.622	65.8%	0.545
D	Negative symptoms	NSA-RS: Restricted speech	2.819	N	0.792	0.583	0.655	0.737	0.663	68.8%	0.500
		NSA-PQ: Poor Quality of Speech	3.359	V	0.846	0.455	0.647	0.714	0.629	65.0%	0.542
		NSA-AB: Affective Blunting	6.180	V	0.609	0.720	0.667	0.667	0.593	66.4%	0.521
		NSA-AM: Amotivation	9.866	VN	0.577	0.773	0.750	0.607	0.599	67.5%	0.542
	Cognitive deficits	BASC-VM: Verbal memory	-0.010	N	0.692	0.773	0.783	0.680	0.642	73.3%	0.542
		BASC-DS: Digit sequencing	0.060	V	0.792	0.542	0.633	0.722	0.669	66.7%	0.500
		BASC-TMT: Token motor task	-0.460	F	0.737	0.783	0.737	0.783	0.843	76.0%	0.548
		BASC-SF: Semantic fluency	0.470	N	0.417	0.792	0.667	0.576	0.571	60.4%	0.500
		BASC-SC: Symbol coding	-0.200	F	0.545	0.900	0.857	0.643	0.631	72.3%	0.524
		BASC-ToL: Tower of London	0.580	B	0.600	0.864	0.800	0.704	0.693	73.2%	0.524
	Psychopathology symptoms	BPRS-AFF: Affective	12.370	V	0.600	0.652	0.652	0.600	0.578	62.6%	0.521
		BPRS-POS: Positive	4.630	VN	0.650	0.929	0.867	0.788	0.751	78.9%	0.583
		BPRS-NEG: Negative	7.210	B	0.421	0.739	0.571	0.607	0.505	58.0%	0.548
		BPRS-RES: Resistance	4.050	N	0.667	0.815	0.737	0.759	0.735	74.1%	0.563
DSH	Negative symptoms	NSA-RS: Restricted speech	2.819	VNF	0.841	0.716	0.732	0.830	0.810	77.8%	0.520
		NSA-PQ: Poor Quality of Speech	2.549	V	0.592	0.814	0.744	0.687	0.733	70.3%	0.523
		NSA-AB: Affective Blunting	5.301	VNF	0.748	0.813	0.798	0.765	0.837	78.0%	0.502
		NSA-AM: Amotivation	8.454	VNF	0.604	0.750	0.705	0.656	0.709	67.7%	0.502
	Cognitive deficits	BASC-VM: Verbal memory	-0.300	VNF	0.699	0.655	0.675	0.679	0.655	67.7%	0.507
		BASC-DS: Digit sequencing	-0.230	VN	0.670	0.644	0.670	0.644	0.625	65.7%	0.519
		BASC-TMT: Token motor task	-0.620	N	0.709	0.655	0.652	0.712	0.672	68.2%	0.523
		BASC-SF: Semantic fluency	-0.270	VN	0.604	0.673	0.640	0.638	0.659	63.8%	0.509
		BASC-SC: Symbol coding	-0.810	VNFB	0.690	0.795	0.777	0.712	0.797	74.2%	0.509
		BASC-ToL: Tower of London	0.260	VNFB	0.712	0.621	0.612	0.720	0.644	66.6%	0.544
	Psychopathology symptoms	BPRS-AFF: Affective	7.470	VN	0.745	0.609	0.648	0.713	0.674	67.7%	0.509
		BPRS-POS: Positive	4.630	VN	0.737	0.658	0.646	0.748	0.735	69.8%	0.542
		BPRS-NEG: Negative	6.040	VNF	0.838	0.717	0.766	0.800	0.780	77.7%	0.525
		BPRS-RES: Resistance	3.970	N	0.544	0.726	0.644	0.636	0.670	63.5%	0.523

Note: Each assessment score is divided into class *Below* (B) and class *Above* (A) by a cut-off threshold (THR). Since we did not find rigorous cut-off scores for factor scales and subscales, the THRs are set as their median or values close to the median such that the counts of both classes are as similar as possible. We computed the majority baseline (MB) of each prediction task as the performance benchmark, in which predictions default to the most frequent class. Best prediction results for verbal (V), non-verbal (N), facial expression (F), and body movement (B) feature sets are presented. Abbreviation: MDD=Major Depressive Disorder, SCZ=Schizophrenia, HCs=Healthy Controls; CM=Confusion Matrix; SEN=Sensitivity; SPE=Specificity; AUPRC=Area Under Precision-Recall Curve; PPV=Positive Predictive Value; NPV=Negative Predictive Value; BAC=Balanced Accuracy.

Supplementary Table 3: Results for automated prediction of NSA-16 scales for schizophrenia (S), depression (D), and healthy controls (H).

Sample	Score: description	THR	Feature	CM			SEN	SPE	PPV	NPV	AUPRC	BAC	MB
					Predicted B	Predicted A							
S	NSA1: Prolonged time to respond	2.00	F	B	25	9							
				A	3	7	0.700	0.735	0.438	0.893	0.803	0.718	0.773
	NSA2: Restricted speech quantity	2.00	N	B	35	10							
				A	11	42	0.792	0.778	0.808	0.761	0.842	0.785	0.541
	NSA3: Impoverished speech content	3.00	V	B	30	17							
				A	22	29	0.569	0.638	0.630	0.577	0.561	0.603	0.520
	NSA6: Reduced modulation of intensity	3.00	VNF	B	33	12							
				A	14	40	0.741	0.733	0.769	0.702	0.703	0.737	0.545
	NSA10: Interest in intimacy	4.00	B	B	34	12							
				A	15	31	0.674	0.739	0.721	0.694	0.725	0.707	0.500
	NSA15: Reduced expressive gestures	3.00	VNF	B	40	14							
				A	14	31	0.689	0.741	0.689	0.741	0.696	0.715	0.545
D	NSA1: Prolonged time to respond	2.00	B	B	27	3							
				A	5	7	0.583	0.900	0.700	0.844	0.799	0.742	0.714
	NSA7: Reduced display on demand	4.00	V	B	23	5							
				A	6	14	0.700	0.821	0.737	0.793	0.794	0.761	0.583
	NSA8: Reduced social drive	4.00	N	B	13	3							
				A	8	24	0.750	0.813	0.889	0.619	0.827	0.781	0.667
	NSA9: Poor rapport with interviewer	2.00	N	B	24	4							
				A	5	15	0.750	0.857	0.789	0.828	0.713	0.804	0.583
	NSA10: Interest in intimacy	3.00	F	B	18	4							
				A	7	13	0.650	0.818	0.765	0.720	0.744	0.734	0.524
	NSA15: Reduced expressive gestures	3.00	VNF	B	24	3							
				A	9	14	0.609	0.889	0.824	0.727	0.736	0.749	0.540
DS	NSA1: Prolonged time to respond	2.00	F	B	48	16							
				A	2	20	0.909	0.750	0.556	0.960	0.818	0.830	0.744
	NSA2: Restricted speech quantity	2.00	VNF	B	54	16							
				A	19	60	0.759	0.771	0.789	0.740	0.810	0.765	0.530
	NSA6: Reduced modulation of intensity	3.00	F	B	31	7							
				A	17	31	0.646	0.816	0.816	0.646	0.688	0.731	0.558
	NSA15: Reduced expressive gestures	3.00	N	B	59	21							
				A	22	44	0.667	0.738	0.677	0.728	0.726	0.702	0.548
	NSA-RS: Restricted Speech	2.82	VNF	B	56	12							
				A	24	57	0.704	0.824	0.826	0.700	0.812	0.764	0.544
	NSA-PQ: Poor Quality of Speech	3.36	VN	B	43	20							
				A	36	47	0.566	0.683	0.701	0.544	0.602	0.624	0.568
	NSA-AB: Affective Blunting	6.26	VNF	B	49	25							
				A	16	59	0.787	0.662	0.702	0.754	0.734	0.724	0.503
	NSA-AM: Amotivation	9.26	VN	B	39	33							
				A	18	56	0.757	0.542	0.629	0.684	0.649	0.649	0.507
	NSA-Total	41.00	F	B	38	6							
				A	16	26	0.619	0.864	0.813	0.704	0.735	0.741	0.512
SH	NSA1: Prolonged time to respond	2.00	VNFB	B	96	42							
				A	10	30	0.750	0.696	0.417	0.906	0.785	0.723	0.775
	NSA2: Restricted speech quantity	2.00	VN	B	58	35							
				A	4	71	0.947	0.624	0.670	0.935	0.850	0.785	0.554
	NSA3: Impoverished speech content	2.00	VNFB	B	64	11							
				A	44	59	0.573	0.853	0.843	0.593	0.705	0.713	0.579
	NSA4: Inarticulate speech	2.00	VN	B	96	27							
				A	8	37	0.822	0.780	0.578	0.923	0.845	0.801	0.732
	NSA6: Reduced modulation of intensity	2.00	F	B	24	10							
				A	11	44	0.800	0.706	0.815	0.686	0.790	0.753	0.618
	NSA14: Reduced daily activity	3.00	VN	B	50	16							
				A	30	72	0.706	0.758	0.818	0.625	0.729	0.732	0.607
	NSA15: Reduced expressive gestures	2.00	VNFB	B	64	10							
				A	29	75	0.721	0.865	0.882	0.688	0.790	0.793	0.584

Supplementary Table 3: Results for automated prediction of NSA-16 scales for schizophrenia (S), depression (D), and healthy controls (H).

Sample	Score: description	THR	Feature	CM	Predicted B	Predicted A	SEN	SPE	PPV	NPV	AUPRC	BAC	MB
SH				B	68	18							
	NSA-RS: Restricted Speech	2.75	VNF	A	22	65	0.747	0.791	0.783	0.756	0.786	0.769	0.503
				B	68	24							
	NSA-PQ: Poor Quality of Speech	2.55	V	A	21	55	0.724	0.739	0.696	0.764	0.744	0.731	0.548
				B	73	17							
	NSA-AB: Affective Blunting	4.66	VNFB	A	25	63	0.716	0.811	0.788	0.745	0.801	0.764	0.506
				B	54	29							
	NSA-AM: Amotivation	7.80	V	A	18	67	0.788	0.651	0.698	0.750	0.768	0.719	0.506
				B	63	18							
	NSA-Total	36.00	VN	A	24	63	0.724	0.778	0.778	0.724	0.790	0.751	0.518
DH				B	55	18							
	NSA2: Restricted speech quantity	2.00	VNFB	A	11	41	0.788	0.753	0.695	0.833	0.760	0.771	0.584
				B	13	4							
	NSA5: Emotion: Reduced range	3.00	N	A	6	19	0.760	0.765	0.826	0.684	0.771	0.762	0.595
				B	37	20							
	NSA6: Reduced modulation of intensity	2.00	N	A	6	55	0.902	0.649	0.733	0.860	0.827	0.775	0.517
				B	68	18							
	NSA9: Poor rapport with interviewer	2.00	VN	A	11	21	0.656	0.791	0.538	0.861	0.775	0.723	0.729
				B	51	14							
	NSA14: Reduced daily activity	3.00	VNFB	A	19	41	0.683	0.785	0.745	0.729	0.708	0.734	0.520
				B	42	15							
	NSA15: Reduced expressive gestures	2.00	VNF	A	17	50	0.746	0.737	0.769	0.712	0.753	0.742	0.540
				B	41	27							
	NSA16: Slowed movements	2.00	B	A	5	35	0.875	0.603	0.565	0.891	0.744	0.739	0.630
				B	50	14							
	NSA-RS: Restricted Speech	2.75	VN	A	13	41	0.759	0.781	0.745	0.794	0.731	0.770	0.542
				B	40	19							
	NSA-PQ: Poor Quality of Speech	2.42	VNFB	A	17	49	0.742	0.678	0.721	0.702	0.691	0.710	0.528
				B	47	15							
	NSA-AB: Affective Blunting	4.21	VNFB	A	9	54	0.857	0.758	0.783	0.839	0.849	0.808	0.504
				B	50	13							
	NSA-AM: Amotivation	6.76	VNFB	A	17	45	0.726	0.794	0.776	0.746	0.754	0.760	0.504
				B	45	13							
	NSA-Total	32.00	VN	A	20	40	0.667	0.776	0.755	0.692	0.703	0.721	0.508
DSH				B	90	32							
	NSA2: Restricted speech quantity	2.00	VNFB	A	21	85	0.802	0.738	0.726	0.811	0.821	0.770	0.535
				B	82	45							
	NSA6: Reduced modulation of intensity	3.00	VN	A	15	74	0.831	0.646	0.622	0.845	0.722	0.739	0.588
				B	109	23							
	NSA8: Reduced social drive	4.00	VNFB	A	37	59	0.615	0.826	0.720	0.747	0.727	0.720	0.579
				B	107	47							
	NSA9: Poor rapport with interviewer	2.00	VN	A	15	47	0.758	0.695	0.500	0.877	0.772	0.726	0.713
				B	94	35							
	NSA14: Reduced daily activity	4.00	VNFB	A	30	69	0.697	0.729	0.663	0.758	0.742	0.713	0.566
				B	68	19							
	NSA15: Reduced expressive gestures	2.00	VNFB	A	35	106	0.752	0.782	0.848	0.660	0.798	0.767	0.618

Note: Each assessment score is divided into class *Below* (B) and class *Above* (A) by a cut-off threshold (THR). The THRs of the NSA-16 scores are set as the median or values close to the median such that the counts of both classes are as similar as possible. Best prediction results for verbal (V), non-verbal (N), facial expression (F), and body movement (B) feature sets are presented. For individual NSA-16 scores, the prediction results with BAC above 70% are presented. We computed the majority baseline (MB) of each prediction task as the performance benchmark, in which predictions default to the most frequent class. Abbreviations: CM=Confusion Matrix; SEN=Sensitivity; SPE=Specificity; AUPRC=Area under precision-recall curve; PPV=Positive Predictive Value; NPV=Negative Predictive Value; BAC=Balanced Accuracy.

Supplementary Table 4: Results for predicting the BACS scales for schizophrenia (S), depression (D), and healthy controls (H).

Sample	Score	THR	Feature	CM	Predicted B	Predicted A	SEN	SPE	PPV	NPV	AUPRC	BAC	MB
DS	BASC-VM: Verbal memory	-0.6	VNF	B	61	9	0.557	0.871	0.830	0.635	0.702	0.714	0.530
				A	35	44							
DS	BASC-DS: Digit sequencing	-0.5	V	B	50	20	0.605	0.714	0.697	0.625	0.673	0.660	0.521
				A	30	46							
DS	BASC-TMT: Token motor task	-0.9	VN	B	54	16	0.632	0.771	0.750	0.659	0.678	0.702	0.521
				A	28	48							
DS	BASC-SF: Semantic fluency	-0.6	VNF	B	50	20	0.658	0.714	0.722	0.649	0.712	0.686	0.530
				A	27	52							
DS	BASC-SC: Symbol coding	-1.1	VNF	B	71	6	0.528	0.922	0.864	0.676	0.728	0.725	0.517
				A	34	38							
DS	BASC-ToL: Tower of London	-0.1	VNF	B	52	13	0.500	0.800	0.764	0.553	0.596	0.650	0.564
				A	42	42							
DS	BASC-Composite	-1.0	VN	B	64	15	0.731	0.810	0.766	0.780	0.805	0.771	0.541
				A	18	49							
DS	BASC-Composite	-2.0	VNFB	B	36	5	0.554	0.878	0.925	0.419	0.787	0.716	0.732
				A	50	62							
SH	BASC-VM: Verbal memory	-0.4	VNF	B	57	28	0.727	0.671	0.696	0.704	0.697	0.699	0.509
				A	24	64							
SH	BASC-DS: Digit sequencing	-0.2	VN	B	51	36	0.691	0.586	0.609	0.671	0.643	0.639	0.518
				A	25	56							
SH	BASC-TMT: Token motor task	-0.8	VNF	B	60	22	0.714	0.732	0.747	0.698	0.748	0.723	0.526
				A	26	65							
SH	BASC-SF: Semantic fluency	-0.5	F	B	23	13	0.736	0.639	0.750	0.622	0.616	0.687	0.596
				A	14	39							
SH	BASC-SC: Symbol coding	-1.0	F	B	31	10	0.667	0.756	0.762	0.660	0.703	0.711	0.539
				A	16	32							
SH	BASC-ToL: Tower of London	0.3	V	B	66	29	0.616	0.695	0.608	0.702	0.613	0.656	0.565
				A	28	45							
SH	BASC-Composite	-1.0	VNF	B	62	17	0.755	0.785	0.807	0.729	0.805	0.770	0.543
				A	23	71							
SH	BASC-Composite	-2.0	VN	B	29	11	0.695	0.725	0.890	0.426	0.820	0.710	0.762
				A	39	89							
DH	BASC-VM: Verbal memory	0.3	VNFB	B	31	34	0.850	0.477	0.600	0.775	0.624	0.663	0.520
				A	9	51							
DH	BASC-DS: Digit sequencing	0.3	VNFB	B	47	21	0.596	0.691	0.618	0.671	0.618	0.644	0.544
				A	23	34							
DH	BASC-TMT: Token motor task	-0.1	VN	B	41	21	0.696	0.661	0.650	0.707	0.665	0.679	0.525
				A	17	39							
DH	BASC-SF: Semantic fluency	0.3	V	B	44	16	0.552	0.733	0.667	0.629	0.607	0.643	0.508
				A	26	32							
DH	BASC-SC: Symbol coding	-0.1	B	B	32	18	0.776	0.640	0.714	0.711	0.686	0.708	0.537
				A	13	45							
DH	BASC-ToL: Tower of London	0.3	VN	B	28	21	0.638	0.571	0.677	0.528	0.563	0.605	0.585
				A	25	44							
DH	BASC-Composite	-1.0	VNFB	B	18	8	0.848	0.692	0.913	0.545	0.788	0.770	0.792
				A	15	84							

Note: The total score of BACS is a composite Z-score, and all BACS subscores are the Z-Scores.[1] Each assessment score is divided into class *Above* (A) and class *Below* (B) by a cut-off threshold (THR). The BACS-Composite THR for normal and mild illness is set to -1.[2] The BACS-Composite THR for mild and severe illness is set to -2.[2] The THRs of other BACS-related scores are set as the median or values close to the median such that the counts of both classes are as similar as possible. Best prediction results for verbal (V), non-verbal (N), facial expression (F), and body movement (B) feature sets are presented. For individual BACS scores, the prediction results with BAC above 70% are presented. We computed the majority baseline (MB) of each prediction task as the performance benchmark, in which predictions default to the most frequent class. Abbreviations: BACS=The Brief Assessment of Cognition in Schizophrenia; CM=Confusion Matrix; SEN=Sensitivity; SPE=Specificity; AUPRC=Area under precision-recall curve; PPV=Positive Predictive Value; NPV=Negative Predictive Value; BAC=Balanced Accuracy.

Supplementary Table 5: Results for predicting the BPRS scales for schizophrenia (S), depression (D), and healthy controls (H).

Sample	Score	THR	Feature	CM	Predicted B	Predicted A	SEN	SPE	PPV	NPV	AUPRC	BAC	MB
S				B	6	3							
	BPRS3: Emotional withdrawal	2.00	F	A	3	32	0.914	0.667	0.914	0.667	0.834	0.790	0.795
				B	15	7							
	BPRS15: Unusual thought content	3.00	F	A	6	16	0.727	0.682	0.696	0.714	0.603	0.705	0.500
				B	45	13							
	BPRS16: Blunted affect	3.00	N	A	13	27	0.675	0.776	0.675	0.776	0.756	0.725	0.592
D				B	31	2							
	BPRS4: Conceptual disorganization	2.00	VN	A	5	10	0.667	0.939	0.833	0.861	0.725	0.803	0.688
				B	19	2							
	BPRS13: Motor retardation	2.00	N	A	12	15	0.556	0.905	0.882	0.613	0.666	0.730	0.563
				B	15	4							
	BPRS16: Blunted affect	2.00	F	A	7	16	0.696	0.789	0.800	0.682	0.658	0.743	0.548
DS				B	72	15							
	BPRS4: Conceptual disorganization	2.00	VN	A	24	35	0.593	0.828	0.700	0.750	0.704	0.710	0.596
				B	69	31							
	BPRS10: Hostility	2.00	VNF	A	14	35	0.714	0.690	0.530	0.831	0.703	0.702	0.671
				B	34	18							
	BPRS16: Blunted affect	3.00	F	A	4	30	0.882	0.654	0.625	0.895	0.744	0.768	0.605
				B	38	35							
	BPRS-AFF: Affective	9.40	V	A	24	49	0.671	0.521	0.583	0.613	0.578	0.596	0.500
				B	30	17							
	BPRS-POS: Positive	5.58	F	A	9	30	0.769	0.638	0.638	0.769	0.732	0.704	0.547
				B	42	30							
	BPRS-NEG: Negative	7.02	N	A	15	59	0.797	0.583	0.663	0.737	0.678	0.690	0.507
				B	47	23							
	BPRS-RES: Resistance	4.62	N	A	40	36	0.474	0.671	0.610	0.540	0.518	0.573	0.521
				B	37	31							
	BPRS-Total	32.00	V	A	21	57	0.731	0.544	0.648	0.638	0.639	0.637	0.534
SH				B	80	18							
	BPRS3: Emotional withdrawal	2.00	VNF	A	28	47	0.627	0.816	0.723	0.741	0.785	0.721	0.566
				B	80	40							
	BPRS13: Motor retardation	2.00	VNF	A	11	42	0.792	0.667	0.512	0.879	0.772	0.730	0.694
				B	83	26							
	BPRS15: Unusual thought content	2.00	VNF	A	15	49	0.766	0.761	0.653	0.847	0.819	0.764	0.630
				B	65	12							
	BPRS16: Blunted affect	2.00	VN	A	29	62	0.681	0.844	0.838	0.691	0.812	0.763	0.542
				B	15	8							
	BPRS-AFF: Affective	12.37	V	A	10	15	0.600	0.652	0.652	0.600	0.578	0.626	0.521
				B	26	2							
	BPRS-POS: Positive	4.63	VN	A	7	13	0.650	0.929	0.867	0.788	0.751	0.789	0.583
				B	17	6							
	BPRS-NEG: Negative	7.21	B	A	11	8	0.421	0.739	0.571	0.607	0.505	0.580	0.548
				B	22	5							
	BPRS-RES: Resistance	4.05	N	A	7	14	0.667	0.815	0.737	0.759	0.735	0.741	0.563
				B	53	20							
	BPRS-Total	24.00	VN	A	22	73	0.768	0.726	0.785	0.707	0.806	0.747	0.565
				B	82	34							
	BPRS-Total	32.00	V	A	11	41	0.788	0.707	0.547	0.882	0.786	0.748	0.690

Supplementary Table 5: Results for predicting the BPRS scales for schizophrenia (S), depression (D), and healthy controls (H).

Sample	Score	THR	Feature	CM	CM Predicted B	CM Predicted A	SEN	SPE	PPV	NPV	AUPRC	BAC	MB
DH	BPRS3: Emotional withdrawal	2.00	VNFB	B<br>A	54<br>14	16<br>41	0.745	0.771	0.719	0.794	0.816	0.758	0.560
	BPRS9: Depressive mood	2.00	N	B<br>A	39<br>13	19<br>47	0.783	0.672	0.712	0.750	0.704	0.728	0.508
	BPRS13: Motor retardation	2.00	F	B<br>A	53<br>6	8<br>20	0.769	0.869	0.714	0.898	0.804	0.819	0.701
	BPRS-AFF: Affective	7.47	VN	B<br>A	67<br>27	43<br>79	0.745	0.609	0.648	0.713	0.674	0.677	0.509
	BPRS-POS: Positive	4.63	VN	B<br>A	77<br>26	40<br>73	0.737	0.658	0.646	0.748	0.735	0.698	0.542
	BPRS-NEG: Negative	6.04	VNF	B<br>A	76<br>19	30<br>98	0.838	0.717	0.766	0.800	0.780	0.777	0.525
	BPRS-RES: Resistance	3.97	N	B<br>A	82<br>47	31<br>56	0.544	0.726	0.644	0.636	0.670	0.635	0.523
	BPRS-Total	24.00	N	B<br>A	57<br>17	8<br>36	0.679	0.877	0.818	0.770	0.789	0.778	0.551
	BPRS-Total	32.00	VN	B<br>A	66<br>6	26<br>20	0.769	0.717	0.435	0.917	0.821	0.743	0.780
DSH	BPRS3: Emotional withdrawal	2.00	VNFB	B<br>A	88<br>39	24<br>77	0.664	0.786	0.762	0.693	0.779	0.725	0.509
	BPRS13: Motor retardation	2.00	VNFB	B<br>A	96<br>18	48<br>66	0.786	0.667	0.579	0.842	0.758	0.726	0.632
	BPRS15: Unusual thought content	2.00	VNF	B<br>A	121<br>22	33<br>47	0.681	0.786	0.588	0.846	0.824	0.733	0.691
	BPRS16: Blunted affect	2.00	VNFB	B<br>A	75<br>34	27<br>92	0.730	0.735	0.773	0.688	0.771	0.733	0.553
	BPRS17: Excitement	2.00	N	B<br>A	129<br>6	58<br>23	0.793	0.690	0.284	0.956	0.868	0.741	0.866

Note: Each assessment score is divided into class *Below* (B) and class *Above* (A) by a cut-off threshold (THR). The THR of BPRS-Total for normal and borderline illness is set to 24.[3] The THR of BPRS-Total for borderline and mild illness is set to 32.[3] The THRs of other BPRS-related scores are set as the median or values close to the median such that the counts of both classes are as similar as possible. Best prediction results for verbal (V), non-verbal (N), facial expression (F), and body movement (B) feature sets are presented. For individual BPRS scores, the prediction results with BAC above 70% are presented. We computed the majority baseline (MB) of each prediction task as the performance benchmark, in which predictions default to the most frequent class. Abbreviations: POS=positive; NEG=Negative; COG=Cognitive; DEP=Depression/Anxiety; DE=Diminished expression; SA=Social amotivation; FSNS=factor score of negative symptoms; CM=Confusion Matrix; SEN=Sensitivity; SPE=Specificity; AUPRC=Area under precision-recall curve; PPV=Positive Predictive Value; NPV=Negative Predictive Value; BAC=Balanced Accuracy.

Supplementary Table 6: Results for predicting the PANSS scale for schizophrenia (S), depression (D), and healthy controls (H).

Sample	Score	THR	Feature	CM			SEN	SPE	PPV	NPV	AUPRC	BAC	MB
					Predicted								
					B	A							
				B	16	8							
	PANSS-POS: Positive factor	7.37	N	A	11	13	0.542	0.667	0.619	0.593	0.573	0.604	0.500
				B	17	6							
	PANSS-NEG: Negative factor	8.45	V	A	6	19	0.760	0.739	0.760	0.739	0.687	0.750	0.521
				B	9	15							
	PANSS-COG: Cognitive factor	5.09	V	A	5	19	0.792	0.375	0.559	0.643	0.512	0.583	0.500
				B	15	7							
	PANSS-DEP: Depression factor	5.55	F	A	9	13	0.591	0.682	0.650	0.625	0.591	0.636	0.500
				B	13	12							
S	PANSS-HOS: Hostility factor	3.08	V	A	5	18	0.783	0.520	0.600	0.722	0.642	0.651	0.521
				B	20	3							
	PANSS-DE: Diminished expression	4.33	V	A	5	20	0.800	0.870	0.870	0.800	0.831	0.835	0.521
				B	20	6							
	PANSS-SA: Social amotivation	5.41	V	A	10	12	0.545	0.769	0.667	0.667	0.647	0.657	0.542
				B	25	9							
	PANSS-FSNS	14.50	V	A	1	13	0.929	0.735	0.591	0.962	0.874	0.832	0.708
				B	14	6							
	PANSS-Total	52.00	VN	A	8	20	0.714	0.700	0.769	0.636	0.688	0.707	0.583
				B	19	4							
	PANSS-POS: Positive factor	3.99	B	A	7	12	0.632	0.826	0.750	0.731	0.766	0.729	0.548
				B	17	8							
	PANSS-NEG: Negative factor	8.01	V	A	10	13	0.565	0.680	0.619	0.630	0.598	0.623	0.521
				B	22	4							
	PANSS-COG: Cognitive factor	4.50	VNF	A	11	13	0.542	0.846	0.765	0.667	0.653	0.694	0.520
				B	9	15							
	PANSS-DEP: Depression factor	8.97	VN	A	6	18	0.750	0.375	0.545	0.600	0.494	0.563	0.500
				B	19	7							
	PANSS-HOS: Hostility factor	3.24	V	A	8	14	0.636	0.731	0.667	0.704	0.642	0.684	0.542
D				B	14	11							
	PANSS-DE: Diminished expression	3.80	F	A	3	14	0.824	0.560	0.560	0.824	0.664	0.692	0.595
				B	17	5							
	PANSS-SA: Social amotivation	5.51	B	A	5	15	0.750	0.773	0.750	0.773	0.724	0.761	0.524
				B	6	2							
	PANSS-FSNS	9.50	VNF	A	5	37	0.881	0.750	0.949	0.545	0.827	0.815	0.840
				B	20	16							
	PANSS-FSNS	14.50	N	A	2	10	0.833	0.556	0.385	0.909	0.711	0.694	0.750
				B	19	7							
	PANSS-Total	52.00	V	A	9	13	0.591	0.731	0.650	0.679	0.592	0.661	0.542
				B	32	10							
	PANSS-POS: Positive factor	4.60	F	A	22	22	0.500	0.762	0.688	0.593	0.637	0.631	0.512
				B	31	17							
	PANSS-NEG: Negative factor	8.08	VN	A	14	34	0.708	0.646	0.667	0.689	0.618	0.677	0.500
				B	33	15							
	PANSS-COG: Cognitive factor	4.93	V	A	10	38	0.792	0.688	0.717	0.767	0.733	0.740	0.500
				B	37	12							
	PANSS-DEP: Depression factor	7.28	V	A	24	23	0.489	0.755	0.657	0.607	0.578	0.622	0.510
				B	35	16							
	PANSS-HOS: Hostility factor	3.08	V	A	17	28	0.622	0.686	0.636	0.673	0.644	0.654	0.531
DS				B	32	16							
	PANSS-DE: Diminished expression	4.18	N	A	8	40	0.833	0.667	0.714	0.800	0.792	0.750	0.500
				B	38	14							
	PANSS-SA: Social amotivation	5.41	V	A	11	33	0.750	0.731	0.702	0.776	0.678	0.740	0.542
				B	8	2							
	PANSS-FSNS	9.50	F	A	21	55	0.724	0.800	0.965	0.276	0.878	0.762	0.884
				B	59	11							
	PANSS-FSNS	14.50	VN	A	10	16	0.615	0.843	0.593	0.855	0.792	0.729	0.729
				B	36	10							
	PANSS-Total	52.00	VN	A	19	31	0.620	0.783	0.756	0.655	0.711	0.701	0.521

Supplementary Table 6: Results for predicting the PANSS scale for schizophrenia (S), depression (D), and healthy controls (H).

Sample	Score	THR	Feature	CM	CM Predicted B	CM Predicted A	SEN	SPE	PPV	NPV	AUPRC	BAC	MB
SH	PANSS-POS: Positive factor	3.99	V	B<br>A	32<br>10	18<br>33	0.767	0.640	0.647	0.762	0.638	0.704	0.538
	PANSS-NEG: Negative factor	6.58	VN	B<br>A	35<br>10	11<br>37	0.787	0.761	0.771	0.778	0.802	0.774	0.505
	PANSS-COG: Cognitive factor	4.50	V	B<br>A	26<br>8	19<br>40	0.833	0.578	0.678	0.765	0.670	0.706	0.516
	PANSS-DEP: Depression factor	4.30	VN	B<br>A	24<br>12	23<br>34	0.739	0.511	0.596	0.667	0.617	0.625	0.505
	PANSS-HOS: Hostility factor	3.08	B	B<br>A	45<br>18	8<br>19	0.514	0.849	0.704	0.714	0.659	0.681	0.589
	PANSS-DE: Diminished expression	3.27	N	B<br>A	34<br>16	6<br>37	0.698	0.850	0.860	0.680	0.841	0.774	0.570
	PANSS-SA: Social amotivation	3.95	V	B<br>A	32<br>16	11<br>34	0.680	0.744	0.756	0.667	0.722	0.712	0.538
	PANSS-FSNS	9.50	VNF	B<br>A	31<br>8	13<br>46	0.852	0.705	0.780	0.795	0.799	0.778	0.551
	PANSS-FSNS	14.50	VN	B<br>A	52<br>0	26<br>15	1.000	0.667	0.366	1.000	0.909	0.833	0.839
	PANSS-Total	38.00	VN	B<br>A	32<br>20	5<br>36	0.643	0.865	0.878	0.615	0.793	0.754	0.602
	PANSS-Total	52.00	V	B<br>A	54<br>9	11<br>19	0.679	0.831	0.633	0.857	0.812	0.755	0.699
DH	PANSS-POS: Positive factor	3.99	F	B<br>A	38<br>7	26<br>16	0.696	0.594	0.381	0.844	0.639	0.645	0.736
	PANSS-NEG: Negative factor	6.45	VNF	B<br>A	43<br>15	6<br>35	0.700	0.878	0.854	0.741	0.771	0.789	0.505
	PANSS-COG: Cognitive factor	4.41	V	B<br>A	31<br>10	14<br>38	0.792	0.689	0.731	0.756	0.737	0.740	0.516
	PANSS-DEP: Depression factor	5.78	VNF	B<br>A	35<br>13	14<br>37	0.740	0.714	0.725	0.729	0.782	0.727	0.505
	PANSS-HOS: Hostility factor	3.08	B	B<br>A	31<br>15	22<br>19	0.559	0.585	0.463	0.674	0.585	0.572	0.609
	PANSS-DE: Diminished expression	3.27	N	B<br>A	32<br>8	16<br>37	0.822	0.667	0.698	0.800	0.782	0.744	0.516
	PANSS-SA: Social amotivation	3.95	VNF	B<br>A	33<br>15	11<br>40	0.727	0.750	0.784	0.688	0.689	0.739	0.556
	PANSS-FSNS	9.50	VNF	B<br>A	43<br>15	6<br>35	0.700	0.878	0.854	0.741	0.771	0.789	0.505
	PANSS-FSNS	14.50	VN	B<br>A	57<br>4	23<br>9	0.692	0.713	0.281	0.934	0.846	0.702	0.860
	PANSS-Total	38.00	VN	B<br>A	31<br>15	7<br>40	0.727	0.816	0.851	0.674	0.812	0.772	0.591
	PANSS-Total	52.00	VNF	B<br>A	62<br>6	14<br>17	0.739	0.816	0.548	0.912	0.835	0.777	0.768
DSH	PANSS-POS: Positive factor	3.99	F	B<br>A	52<br>28	21<br>30	0.517	0.712	0.588	0.650	0.621	0.615	0.557
	PANSS-NEG: Negative factor	7.16	VN	B<br>A	49<br>16	21<br>55	0.775	0.700	0.724	0.754	0.734	0.737	0.504
	PANSS-COG: Cognitive factor	4.48	V	B<br>A	47<br>27	22<br>45	0.625	0.681	0.672	0.635	0.682	0.653	0.511
	PANSS-DEP: Depression factor	5.78	VNFB	B<br>A	57<br>30	17<br>44	0.595	0.770	0.721	0.655	0.689	0.682	0.500
	PANSS-HOS: Hostility factor	3.08	B	B<br>A	49<br>24	27<br>32	0.571	0.645	0.542	0.671	0.554	0.608	0.576
	PANSS-DE: Diminished expression	3.51	VNFB	B<br>A	56<br>18	20<br>54	0.750	0.737	0.730	0.757	0.795	0.743	0.514
	PANSS-SA: Social amotivation	4.03	VNFB	B<br>A	59<br>29	16<br>44	0.603	0.787	0.733	0.670	0.697	0.695	0.507

Supplementary Table 6: Results for predicting the PANSS scale for schizophrenia (S), depression (D), and healthy controls (H).

Sample	Score	THR	Feature	CM	Predicted B	Predicted A	SEN	SPE	PPV	NPV	AUPRC	BAC	MB
DSH	PANSS-FSNS	9.50	VNF	B	43	9							
				A	26	70	0.729	0.827	0.886	0.623	0.808	0.778	0.649
	PANSS-FSNS	14.50	VN	B	97	17							
				A	10	17	0.630	0.851	0.500	0.907	0.850	0.740	0.809
	PANSS-Total	38.00	VN	B	31	8							
				A	30	72	0.706	0.795	0.900	0.508	0.810	0.750	0.723
	PANSS-Total	52.00	V	B	65	26							
				A	11	39	0.780	0.714	0.600	0.855	0.758	0.747	0.645

Note: Each assessment score is divided into class *Above* (A) and class *Below* (B) by a cut-off threshold (THR). The THR of PANSS-Total and PANSS-FSNS for normal and borderline illness is set to 38 and 9.5, respectively.[4,5] The PANSS-Total THR for borderline and mild illness is set to 52 and 14.5, respectively.[4,5] The THRs of other PANSS-related scores are set as the median or values close to the median such that the counts of both classes are as similar as possible. Best prediction results for verbal (V), non-verbal (N), facial expression (F), and body movement (B) feature sets are presented. For individual PANSS scores, the prediction results with BAC above 70% are presented. We computed the majority baseline (MB) of each prediction task as the performance benchmark, in which predictions default to the most frequent class. Abbreviations: POS=positive; NEG=Negative; COG=Cognitive; DEP=Depression/Anxiety; DE=Diminished expression; SA=Social amotivation; FSNS=factor score of negative symptoms; CM=Confusion Matrix; SEN=Sensitivity; SPE=Specificity; AUPRC=Area under precision-recall curve; PPV=Positive Predictive Value; NPV=Negative Predictive Value; BAC=Balanced Accuracy.

Supplementary Table 7: Results for automated classification of schizophrenia (S), depression (D), and healthy controls (H).

Task	Feature	CM	Predicted D	Predicted H	SEN	SPE	PPV	NPV	AUPRC	BAC	MB
D vs. H	V	D	35	13	0.729	0.729	0.648	0.797	0.799	0.729	0.593
		H	19	51							
	N	D	39	9	0.813	0.771	0.709	0.857	0.817	0.792	0.593
		H	16	54							
	F	D	23	19	0.548	0.778	0.697	0.648	0.714	0.663	0.517
		H	10	35							
	B	D	29	13	0.690	0.682	0.580	0.776	0.696	0.686	0.611
		H	21	45							
	VN	D	41	7	0.854	0.757	0.707	0.883	0.861	0.806	0.593
		H	17	53							
	VNF	D	41	9	0.820	0.784	0.719	0.866	0.865	0.802	0.597
		H	16	58							
	VNFB	D	37	13	0.740	0.907	0.841	0.840	0.879	0.823	0.600
		H	7	68							
			**H**	**S**	**SEN**	**SPE**	**PPV**	**NPV**	**AUPRC**	**BAC**	**MB**
S vs. H	V	H	49	21	0.867	0.700	0.802	0.790	0.847	0.784	0.583
		S	13	85							
	N	H	50	20	0.827	0.714	0.802	0.746	0.777	0.770	0.583
		S	17	81							
	F	H	31	14	0.795	0.689	0.714	0.775	0.795	0.742	0.506
		S	9	35							
	B	H	40	26	0.696	0.606	0.711	0.588	0.698	0.651	0.582
		S	28	64							
	VN	H	51	19	0.888	0.729	0.821	0.823	0.866	0.808	0.583
		S	11	87							
	VNF	H	52	22	0.939	0.703	0.809	0.897	0.873	0.821	0.572
		S	6	93							
	VNFB	H	55	20	0.913	0.733	0.825	0.859	0.889	0.823	0.579
		S	9	94							
			**D**	**S**	**SEN**	**SPE**	**PPV**	**NPV**	**AUPRC**	**BAC**	**MB**
D vs. S	V	D	36	12	0.694	0.750	0.850	0.545	0.788	0.722	0.671
		S	30	68							
	N	D	39	9	0.755	0.813	0.892	0.619	0.857	0.784	0.671
		S	24	74							
	F	D	30	12	0.773	0.714	0.739	0.750	0.774	0.744	0.512
		S	10	34							
	B	D	30	12	0.707	0.714	0.844	0.526	0.793	0.710	0.687
		S	27	65							
	VN	D	37	11	0.837	0.771	0.882	0.698	0.858	0.804	0.671
		S	16	82							
	VNF	D	43	7	0.808	0.860	0.920	0.694	0.893	0.834	0.664
		S	19	80							
	VNFB	D	41	9	0.874	0.820	0.909	0.759	0.905	0.847	0.673
		S	13	90							

Supplementary Table 7: Results for automated classification of schizophrenia (S), depression (D), and healthy controls (H).

Task	Feature		CM			SEN	SPE	PPV	NPV	AUPRC	BAC	MB
				Predicted								
				H	P							
DS vs. H	V	H		47	23							
		P		26	120	0.671	0.822	0.644	0.839	0.826	0.747	0.676
	N	H		56	14							
		P		45	101	0.800	0.692	0.554	0.878	0.795	0.746	0.676
	F	H		34	11							
		P		25	61	0.756	0.709	0.576	0.847	0.772	0.732	0.656
	B	H		31	35							
		P		34	99	0.470	0.744	0.477	0.739	0.682	0.607	0.668
	VN	H		57	13							
		P		37	109	0.814	0.747	0.606	0.893	0.867	0.780	0.676
	VNF	H		56	18							
		P		27	122	0.757	0.819	0.675	0.871	0.854	0.788	0.668
	VNFB	H		58	17							
		P		27	125	0.773	0.822	0.682	0.880	0.861	0.798	0.670
			D	H	S	SEN	SPE	PPV	NPV	AUPRC	BAC	MB
D vs. S vs. H	V	D	31	11	6							
		H	16	44	10	0.541	0.864	0.768	0.694	0.691	0.605	0.454
		S	30	15	53							
	N	D	29	9	10							
		H	14	41	15	0.643	0.788	0.716	0.727	0.654	0.611	0.454
		S	17	18	63							
	F	D	21	8	13							
		H	6	25	14	0.568	0.690	0.481	0.759	0.615	0.541	0.344
		S	11	8	25							
	B	D	18	13	11							
		H	15	29	22	0.511	0.694	0.588	0.625	0.556	0.460	0.460
		S	18	27	47							
	VN	D	39	6	3							
		H	12	45	13	0.633	0.864	0.795	0.739	0.742	0.696	0.454
		S	24	12	62							
	VNF	D	34	11	5							
		H	11	48	15	0.657	0.839	0.765	0.754	0.762	0.662	0.444
		S	21	13	65							
	VNFB	D	35	10	5							
		H	9	51	15	0.680	0.840	0.778	0.761	0.780	0.687	0.452
		S	19	14	70							

Note: We report here the classification results for various choices of features sets, i.e., verbal (V), non-verbal (N), facial expression (F), body movement (B), speech (VN), facial and speech (VNF), and all feature sets combined (VNFB). We computed the majority baseline (MB) of each prediction task as the performance benchmark, in which predictions default to the most frequent class. Abbreviations: P=Patient; CM=Confusion Matrix; SEN=Sensitivity; SPE=Specificity; AUPRC=Area under precision-recall curve; PPV=Positive Predictive Value; NPV=Negative Predictive Value; BAC=Balanced Accuracy.

Supplementary Table 8: Overview of related behavioral data-driven studies on predicting the severity of negative and general psychiatric symptoms of schizophrenia and depression.

Sample	Aspect	Reference	Year	Dataset	Data Source	Computational Features	Classifier	Assessment Scale	Performance (ACC/Pearson's R/MAE/RMSE)
SCZ	Verbal (spoken language)	Xu et al.[6]	2018	SCZ = 50 (25M, 25F); HCs = 25 (11M, 14F)	Kaldi's transcriptions of semi-structured clinical interview	LIWC, DICTION7.0, Doc2Vec	Ensemble classifier	NSA-16	ACC: NSA-Prolonged time to respond=82.69%; NSA-Restricted speech quantity=82.69%; NSA-Impoverished speech content=80.77%; NSA-Emotion reduced range=61.54%; NSA-Reduced modeulation of intensity=78.85%; NSA-Reduced expressive gestures=84.62%
	Non-verbal (acoustic/ prosodic/ conversational)	Cohen et al.[7]	2020	121 patients (SCZ=76, MDD=18, BD=20, others=7)	Audio recordings of picture task and free speech for daily routines	Acoustic features	LASSO	BPRS and SANS	SANS-Alogia: ACC=85% to 87%, SEN=0.60 to 0.70, SPE=0.92 to 0.93; SANS-Blunted Vocal Affect: ACC=89% to 96%, SEN=0.66 to 0.82, SEN=0.92 to 0.99
		Chakraborty et al.[8]	2018	SCZ = 52 (25M, 27F); HCs = 26 (12M, 14F)	Audio recordings of semi-structured clinical interview	Acoustic/prosodic features	KNN, SVM, and AdaBoost	NSA-16	ACC: NSA-Prolonged time to respond=82.69%; NSA-Restricted speech quantity=82.69%; NSA-Impoverished speech content=80.77%; NSA-Emotion reduced range=61.54%; NSA-Reduced modulation of intensity=78.85%; NSA-Reduced expressive gestures=84.62%
		Wörtwein et al.[9]	2017	20 psychiatric individuals with SCZ, MDD, or mania	Audio recordings of semi-structured clinical interview	Acoustic features	SVR	BPRS-24	Pearson' R/MAE: BPRS-Elevated mood=0.68/0.80; BPRS-Grandiosity=0.80/0.46; BPRS-Excitement=0.72/0.69; BPRS-Motor hyperactivity=0.67/0.84; BPRS-Total=0.49/10.35
	Facial expressions	Bishay et al.[10]	2019	91 out-patients	Video recordings of body psychotherapy of schizophrenia	Facial expressions	DNN-GMM	PANSS	PANSS-NEG: MAE = 3.35 and RMSE=4.27
		Tron et al.[11]	2016	SCZ = 34; HCs = 33	Video recordings of structured interview	Facial cluster features	Ridge regression	PANSS	Pearson's R between predictions and scales: PANSS-Blunted affect=0.431
		Tron et al.[12]	2015	SCZ = 34; HCs = 33	Video recordings of structured interview	Facial AUs	Ridge regression	PANSS	Pearson's R between predictions and scales: PANSS-Blunted affect=0.530; PANSS-Emotional withdrawal=0.510; PANSS-Difficulty in abstract thinking =0.369; PANSS-Stereotyped thinking =0.369; PANSS-Passive withdrawal=0.368
	Body movement	Chakraborty et al.[13]	2017	SCZ = 46 (23M, 23F); HCs = 23 (11M, 12F)	Kinect recording of semi-structured clinical interview	Linear Speed and Accelaration of Body Joints	KNN and SVM	NSA-16	ACC: NSA-Prolonged time to respond=60.87%; NSA-Restricted speech quantity=82.69%%; NSA-Impoverished speech content=67.39%; NSA-Emotion reduced range=61.54%; NSA-Reduced modeulation of intensity=63.04%

Supplementary Table 8: Overview of related behavioral data-driven studies on predicting the severity of negative and general psychiatric symptoms of schizophrenia and depression.

Sample	Aspect	Reference	Year	Dataset	Data Source	Computational Features	Classifier	Assessment Scale	Performance (ACC/Pearson's R/MAE/RMSE)
SCZ	Multiple modalities	Xu et al.[14]	2019	SCZ = 43 (21M, 22F); MDD = 45 (23F,22F) HCs = 41 (23M, 18F)	Audio recordings of semi-structured clinical interview	Verbal: linguistic features Audio: acoustic/prosodic features	Ensemble classifier	NSA-16	ACC: NSA-Restricted speech quantity=90.5%; NSA-Reduced Display=71.4%; NSA-Reduced expressive gestures=64.3%
SCZ & MDD	Non-verbal (acoustic/ prosodic/ conversational)	Cohen AS, et al.[7]	2020	121 patients (SCZ=76, MDD=18, BD=20, others=7)	Audio recordings of picture task and free speech for daily routines	Acoustic features	LASSO	BPRS and SANS	SANS-Alogia: ACC=85% to 87%, SEN=0.60 to 0.70, SPE=0.92 to 0.93; SANS-Blunted Vocal Affect: ACC=89% to 96%, SEN=0.66 to 0.82, SEN=0.92 to 0.99
		Wörtwein et al.[9]	2017	20 psychiatric individuals with SCZ, MDD, or mania	Audio recordings of semi-structured clinical interview	Acoustic features	SVR	BPRS-24	Pearson' R/MAE: BPRS-Elevated mood=0.68/0.80; BPRS-Grandiosity=0.80/0.46; BPRS-Excitement=0.72/0.69; BPRS-Motor hyperactivity=0.67/0.84; BPRS-Total=0.49/10.35
	Multiple Modalities	Xu S, et al.[14]	2019	SCZ = 43 (21M, 22F); MDD = 45 (23F,22F) HCs = 41 (23M, 18F)	Audio recordings of semi-structured clinical interview	Verbal: linguistic features Audio: acoustic/prosodic features	Voting Classifier	NSA-16	ACC: NSA-Impoverished speech content=70.5%; NSA-Emotion reduced range=70.5%; NSA-Reduced sexual interest=68.2%; NSA-Reduced expressive gestures=70.5%

Abbreviation: SCZ=Patient with schizophrenia, HCs=Healthy controls, MDD=Depression patient, BD=Bipolar disorder, NSA-16=The 16-item Negative Symptom Assessment, BPRS=The Brief Psychiatric Rating Scale, PANSS=The Positive and Negative Syndrome Scale, AUs=Action units, LR=Logistic Regression, RF=Random Forest, SVM=Support Vector Machines, KNN=K-Nearest Neighbors, SVR=Support Vector Regression, ACC=Accuracy, BAC=Balanced accuracy, AUC=Area under ROC curve, F1=F1 score, MAE=Mean absolute error, RMSE=Root mean square error, CV=Cross-validation.

Supplementary Table 9: Overview of related behavioral data-driven studies on diagnosis of schizophrenia and depression.

Task	Aspect	Reference	Year	Dataset	Data Source	Computational Features	Classifier	Performance (ACC/BAC/AUC)
SCZ vs. HCs	Verbal (spoken language)	Hong et al,[15]	2015	SCZ = 23 (12M, 11F); HCs = 16 (7M, 9F)	Manual transcriptions of emotional narrative	Lexical features	SVM	ACC = 74.4% (with CV)
		Elvevåg B, et al.[16]	2007	SCZ = 26 (19M, 7F); HCs = 25 (10M, 15F)	Manual transcriptions of clinical interview	LSA-based features	LDA	ACC=78.4% (with CV)
		Elvevåg et al.[17]	2010	SCZ = 53 (19M, 7F); HCs = 30 (10M, 15F)	Manual transcriptions of clinical interview	LSA-based features	LDA	ACC=77.1% (with CV)
		Corcoran et al.[18]	2018	SCZ (CHR+) = 19 (17M, 2F); HCs = 21 (13M, 8F)	Manual transcriptions of Caplan's "Story Game"	LSA-based features	LR	ACC=72% (with CV)
		Bar et al.[19]	2019	SCZ = 24; HCs = 27 (All Male)	Manual transcriptions of 18 clinical questions	Semantic features	RF, XGB, and SVM	ACC=70.4 to 81.5% (with CV)
		Mota et al.[20]	2012	SCZ = 8 (7M, 1F); HCs = 8 (5M, 2F); Manic = 8 (8M, 0F)	Manual transcriptions of the description of a recent dream	Graph-based features	NB, SVM, DT, MLP, and RBF	AUC=50 to 90% (with CV)
		Rezaii et al.[21]	2019	Training set: SCZ = 7 (4M, 3F); HCs = 23 (10M, 15F) Testing set: SCZ = 5 (19M, 7F); HCs = 5 (10M, 15F)	Manual transcriptions of structured interview	Word2Vec-based semantic density	NA	Training: ACC=93%; Testing: ACC=90%
		Xu et al.[6]	2018	SCZ = 50 (25M, 25F); HCs = 25 (11M, 14F)	Kaldi automated transcriptions of semi-structured clinical interview	LIWC, DICTION7.0, Doc2Vec	Ensemble classifier	ACC=78.7% (with CV)
		Xu et al.[22]	2019	SCZ = 47 (22M, 25F); HCs = 24 (10M, 14F)	Google ASR automated transcriptions of semi-structured clinical interview	LIWC	SVM, LR, KNN	ACC=84.5 to 85.9% (with CV)
		Voppel et al.[23]	2021	SCZ = 50 (12M, 8F); HCs = 50 (6M, 5F)	Audio recordings of semi-structured clinical interview	Word2Vec-based language connectedness	RF	ACC=85% (with CV)
		Tang et al.[24]	2021	SCZ = 20 (11M, 9F); HCs = 11 (4M, 7F)	Manual transcriptions of semi-structured clinical interview	BERT-derived features	NB	ACC = 87%, AUC=0.91 (with CV)
	Non-verbal (acoustic/ prosodic/ conversational)	Kliper et al.[25]	2019	SCZ = 22; MDD = 20; HCs = 20	NA	Acoustic features	Linear Classifier	SCZ vs. HCs: ACC=73.8 to 81.0% MDD vs. HCs: ACC=69.0 to 87.1% SCZ vs. MDD: ACC=52.0 to 76.7% (all with CV)
		Espinola et al.[25]	2021	SCZ = 20 (12M, 8F); HCs = 11 (6M, 5F)	Audio recordings of routine medical assessment	Acoustic features	SVM,LR,NB, RF, MLP, and DT	ACC=63.97% to 91.76% (with CV)
		Chakraborty et al.[8]	2018	SCZ = 52 (25M, 27F); HCs = 26 (12M, 14F)	Audio recordings of semi-structured clinical interview	Acoustic/prosodic features	Linear SVM	ACC = 79.49% (with CV)
		Tahir et al.[26]	2016	SCZ = 8 (4M, 4F); HCs = 7 (2M, 5F)	Audio recordings of semi-structured clinical interview	Conversational features	SVM and SVR	ACC = 86.0 to 93.0% (with CV)
		Martínez-Sánchez et al.[27]	2015	SCZ = 45 (32M, 13F); HCs = 35 (22M, 13F)	Audio recordings of semi-structured clinical interview	Acoustic/prosodic features	LDA	ACC = 87.5% (with CV)

Supplementary Table 9: Overview of related behavioral data-driven studies on diagnosis of schizophrenia and depression.

Task	Aspect	Reference	Year	Dataset	Data Source	Computational Features	Classifier	Performance (ACC/BAC/AUC)
SCZ vs. HCs	Non-verbal	Rapcan et al.[28]	2010	SCZ = 39 (32M, 13F); HCs = 18 (22M, 13F)	Digitally recordings of reading aloud a text passage	Acoustic features	LDA	ACC = 79.4% (with CV)
	Facial expressions	Tron et al.[11]	2016	SCZ = 34; HCs = 33	Video recordings of structured interview	Facial cluster features	SVM	AUC = 0.80 to 0.85 (with CV)
		Tron et al.[12]	2015	SCZ = 34; HCs = 33	Video recordings of structured interview	Facial AUs	SVM	AUC = 0.80 (with CV)
	Body movement	Chakraborty et al.[13]	2017	SCZ = 46 (23M, 23F); HCs = 23 (11M, 12F)	Kinect recording of semi-structured clinical interview	linear speed and acceleration of body joints	MLP	ACC=86.76%; AUC=0.913 (with CV)
	Multiple modalities	Xu et al.[14]	2019	SCZ = 43 (21M, 22F); MDD = 45 (23F,22F) HCs = 41 (23M, 18F)	Audio recordings of semi-structured clinical interview	Verbal: linguistic features Audio: acoustic/prosodic features	Ensemble classifier	SCZ vs. HCs: ACC=76.19% MDD vs. HCs: ACC=72.09% SCZ vs. MDD: ACC=69.32% (all with CV)
MDD vs. HCs	Verbal (spoken language)	Qureshi et al.[29]	2020	DAIC dataset: 138 samples split into 5 classes	interview transcriptions of human-agent interaction	Sentence encoding network	LSTM	ACC=66%, F1=0.60 (5-class classification, with CV)
	Non-verbal (acoustic/ prosodic/ conversational)	Scherer et al.[30]	2013	DAIC dataset: MDD-severe = 18; MDD-low = 18	Audio and video recordings of human-computer interaction experiment	Acoustic features	SVM	F1-avg=0.738; ACC=75%
		Harati et al.[31]	2021	Ellipsis dataset: 12872 sessions from 10,932 speakers (6450F and 4482M)	Audio recordings of response of open ended questions.	Acoustic features	CNN/LSTM	PHQ-8<10 vs. PHQ-8≥10: AUC=0.79 (on testing set)
		Espinola et al.[32]	2021	MDD = 22 (17M, 5F); HCs = 11(6M, 5F)	Audio recordings of medical evaluation interview	Acoustic features	Various Classifiers	ACC=60% to 89.14%
		Low et al.[33]	2011	MDD = 68 (19M, 49F); HCs = 71 (27M, 44F) (between 14 and 18 years old)	Video recordings of family interation	Acoustic features	GMM-SVM	ACC = 67-87% (with CV)
		Ooi et al.[34]	2013	MDD (at risk) = 15 (6M, 9F); HCs (no risk) = 15 (6M, 9F) (all adolescent, age 12 to 13 years)	Audio recordings of child-adult interaction	Acoustic Features	GMM-Bayesian classifier	ACC = 73% (with CV)
		Huang et al.[35]	2020	SH2-FS dataset: Train: MDD = 97; HCs = 364 Test: DF=23; HCs=105 DAIC dataset: Train: 107 Test: 35	Audio recordings of free speech in naturalistic environments	Acoustic Features	CNN	DAIC dataset: BAC=91%, F1-avg=0.915; SH2-FS: BAC=73%, F1-avg=0.625
		Sanchez et al.[36]	2011	MDD-severe = 16; MDD-low = 16	Audio recordings of structured clinical interview	Prosodic and spectral features	SVM	ACC = 81.3% (with CV)
		Taguchi T[37]	2015	MDD = 36 (22M, 14F); HCs = 36(16M, 20F)	Audio recordings of reading out numbers and verbal fluency task	Second dimension of MFCC	SDA	ACC = 81.9% (no CV)

Supplementary Table 9: Overview of related behavioral data-driven studies on diagnosis of schizophrenia and depression.

Task	Aspect	Reference	Year	Dataset	Data Source	Computational Features	Classifier	Performance (ACC/BAC/AUC)
MDD vs. HCs	Facial expressions	Dibeklioğlu H, et al.[38]	2018	Pitt dataset: MDD-severe = 58; MDD-mild = 35; MDD-low =37 (multi-session data)	Audio and video recordings of HRSD clinical interview.	Facial movement	SDAE	ACC=72.6% (with CV)
		Cohn et al.[39]	2009	Pitt dataset: MDD-severe = 66; MDD-low =41 (multi-session data)	Audio and video recordings of HRSD clinical interview.	Facial movement	SVM	ACC=79% (with CV)
	Body movement	Horigome et al.[40]	2020	MDD = 17 (8M, 9F); BD = 14 (7M,7F); HCs = 16(6M, 10F)	Kinect RGB+D recording of semi-structured clinical interview	Body movement featrues	SVM-rbf	ACC=72% (with CV)
	Multiple modalities	Joshi et al.[41]	2013	BlackDog dataset: MDD = 30; HCs = 30	Audio and video recordings of clinical interview	Audio: acoustic features; Video: Spatio-temporal descriptor	SVM	Audio: ACC = 78.3 to 83.3%; Video: ACC = 78.8 to 81.7%; Audio + Video = 66.7 to 91.7% (no CV and obtained by fine tuning the parameters)
		Lu et al.[42]	2020	Ellipsis dataset: 16000 sessions from about 11000 speakers	Audio recordings of response of open ended questions.	Acoustic model and NLP model	Acoustic: CNN/LSTM NLP: ULMFiT	PHQ-8¡10 vs. PHQ-810: Acoustic: AUC=0.803 NLP: AUC=0.830
		Yang et al.[43]	2016	DAIC dataset: MDD-severe = 7; MDD-low = 28	Audio and video recordings of human-agent interaction	Facial expression, facial movement features	SVM	Test Set: F1=0.724; BAC=75.4%;
		Alghowinem et al.[44]	2015	BlackDog dataset: MDD = 30; HCs = 30(30M, 30F) Pitt dataset: MDD-severe = 19; HCs(Symptom-free) = 19(14M, 24F) DAIC dataset: MDD-severe = 16; MDD-low = 16 (9M, 23F)	BlackDog dataset: Audio and video recordings of open-ended questions interview. Pitt dataset: Audio and video recordings of HRSD clinical interview. DAIC dataset: Audio/Video recordings of human-agent interaction	Eye activity and head pose	SVM	BlackDog: ACC=76.7% Pitt: ACC=94.7% DAIC: ACC=68.8% All three combined: ACC=73.1 (with CV)
		Dibeklioğlu et al.[45]	2015	Pitt dataset: MDD-severe = 58; MDD-low = 37 (multi-session data)	Audio and video recordings of HRSD clinical interview.	Facial movement, head movement, and Audio	LR	Facial: ACC=81.44% Head: ACC=79.59% Audio: ACC=69.73% All three combined: ACC=88.93% (with CV)
		Valstar et al.[46]	2016	DAIC-WOZ (NA)	Audio/Video recordings of human-agent interaction	Audio: acoustic features; Video: facial and eye expressions	SVM	Test data: Audio: F1-avg = 0.50; Video: F1-avg = 0.72; Audio + Video: F1-avg = 0.72
		Alghowinem et al.[47]	2018	BlackDog dataset: MDD = 30; HCs = 30	Audio and video recordings of open-ended questions interview.	Audio: acoustic and conversational features; Video: Eye and heald movement features	SVM	Audio: BAC = 81.7%; Video: BAC = 63.3 to 78.3%; Audio + Video = 63.3 to 86.7% (with CV)

Supplementary Table 9: Overview of related behavioral data-driven studies on diagnosis of schizophrenia and depression.

Task	Aspect	Reference	Year	Dataset	Data Source	Computational Features	Classifier	Performance (ACC/BAC/AUC)
MDD vs. HCs	Multiple modalities	Joshi et al.[48]	2013	BlackDog dataset: MDD = 30; HCs = 30	Audio and video recordings of open-ended questions interview.	Facial and upper body movement	SVM	Facial: ACC=71%, F1 = 0.73; Body movement: ACC=77%, F1=0.8 (with CV)
MDD vs. SCZ	Verbal (spoken language)	Lott et al.[49]	2002	SCZ = 47 (33M, 14F); BD = 29 (17M, 12F); MDD = 23 (10M, 13F)	Manual transcriptions of the structured clinical interview	Linguistic features	LDA	SCZ: Recall=74.1%; BD: Recall=63.3%; MDD: Recall=82.4%; Three-class classification: ACC=72.7% (with CV)
	Non-verbal (acoustic/ prosodic/ conversational)	Kliper et al.[25]	2010	SCZ = 22; MDD = 20 HCs = 20	NA	Acoustic features	Linear Classifier	SCZ vs. HCs: ACC=73.8 to 81.0% MDD vs. HCs: ACC=69.0 to 87.1% SCZ vs. MDD: ACC=52.0 to 76.7% (with CV)

Abbreviation: SCZ=Patient with schizophrenia, HCs=Healthy controls, MDD=Patient with major depression disorder, BD=Bipolar disorder, TLC=the Assessment of Thought, Language and Communication, BPRS=the Brief Psychiatric Rating Scale, AUs=Action units, LDA=Linear Discriminant Analysis, LSA=Latent Semantic Analysis, LR=Logistic Regression, RF=Random Forest, SVM=Support Vector Machines, XGB=XGBoost, KNN=K-Nearest Neighbors, SVR=Support Vector Regression, LIWC=Linguistic Inquiry and Word Count, ASR=Automatic speech recognition, MLP=Multilayer Perceptron, HDRS=Hamilton Depression Rating Scale, BDI=Beck's Depression Inventory, SDA=Stepwise discriminant analysis, HRSD=Hamilton Rating Scale for Depression, SDAE=Stacked denoising auto-encoders, ACC=Accuracy, BAC=Balanced accuracy, AUC=Area under ROC curve, F1=F1 score, MAE=Mean absolute error, RMSE=Root mean square error, CV=Cross-validation, DAIC=the Distress Analysis Interview Corpus, LSTM=Long Short Term Memory, CNN=Convolutional Neural Network, ULMFiT=Universal Language Model Fine-tuning.

Supplementary Table 10: Low-level descriptors (LLDs) in the OpenSMILE and DisVoice toolkits.

LLDs Name (Abbreviation)	Description
Intensity/Energy[a,b]	Power carried by the audio waves
Loudness[a]	Normalised intensity raised to the power of 0.3
Mel-frequency cepstral coefficients (MFCCs)[a,b]	12 Mel-frequency cepstral coefficients from 25 ms audio frames with 10ms sliding window
Pitch (F0)[a,b]	Fundamental frequency computed from the cepstrum
Probability of voice (VoiceProb)[a]	Voicing probability computed from the auto-correlation function (ACF)
F0 envelope (F0env)[a]	Envelope of the smoothed fundamental frequency
Line spectral frequencies (LSF1-8)[a]	Line spectral frequencies computed from 8 linear predictive coefficients
Zero crossing rate (ZCR)[a]	Zero-crossing rate of time signal (frame-based)
First formant frequency (FF1)[b]	Frequency of the first formant
Second formant frequency (FF2)[b]	Frequency of the second formant
22 Bark band energies (BBEs)[b]	Spectral energy over the 1-22 bark scales
Voiced/Pause duration (VoiceDur/PauseDur)[b]	Duration of voiced segments/pauses
Voiced/Pause rate (VoicedRate/PauseRate)[b]	Number of voiced segments/pauses per second
Logaritmic energy (LogE)[b]	Logarithmic scale of acoustical power
Jitter[b]	Average absolute difference between the frequency of consecutive periods
Shimmer[b]	Average absolute difference between the amplitudes of consecutive periods
Amplitude perturbation quotient (APQ)[b]	Average difference between the amplitude of five preceding and successive pitch periods
Pitch perturbation quotient (PPQ)[b]	Variability of the pitch period evaluated in five consecutive cycles

[a] Evaluated by OpenSMILE.
[b] Evaluated by DisVoice.

Supplementary Table 11: Key hyperparameters of all five base classifiers in the ensemble classifier.

Classifier	Parameter name	Value	Description of parameter
Linear SVM	C	1.0	Regularization parameter
	class_weight	balanced	Weights of each class are automatically adjusted as n_samples/(n_classes*n_samples_in_class)
	max_iter	100000	Hard limit of 100000 iterations
Logistic Regression	solver	L-BFGS	L-BFGS is an optimization algorithm that approximates the Broyden–Fletcher–Goldfarb –Shanno algorithm (BFGS) using a limited amount of computer memory
	penalty	L2	L2 regularization
	C	1.0	Regularization parameter
	class_weight	balanced	Weights of each class are automatically adjusted as n_samples/(n_classes*n_samples_in_class)
	max_iter	100000	Hard limit of 100000 iterations
Gradient Boosting	loss	deviance	Use binomial deviance loss for classification with probabilistic outputs
	learning_rate	0.1	Learning rate shrinks the contribution of each tree
	n_estimators	100	The number of boosting stages to perform
	max_depth	3	Maximum depth of the individual regression estimators
AdaBoost	n_estimators	100	The number of boosting stages to perform
	algorithm	SAMME.R	Real Stagewise Additive Modeling using a Multi-class Exponential (SAMME.R) loss function
	learning_rate	1.0	Learning rate shrinks the contribution of each tree
Random Forest	n_estimators	400	The number of trees in the forest
	criterion	Gini	The Gini impurity is used to measure the quality of a split
	class_weight	balanced	Weights of each class are automatically adjusted as n_samples/(n_classes*n_samples_in_class)
	max_depth	7	The maximum depth of the tree

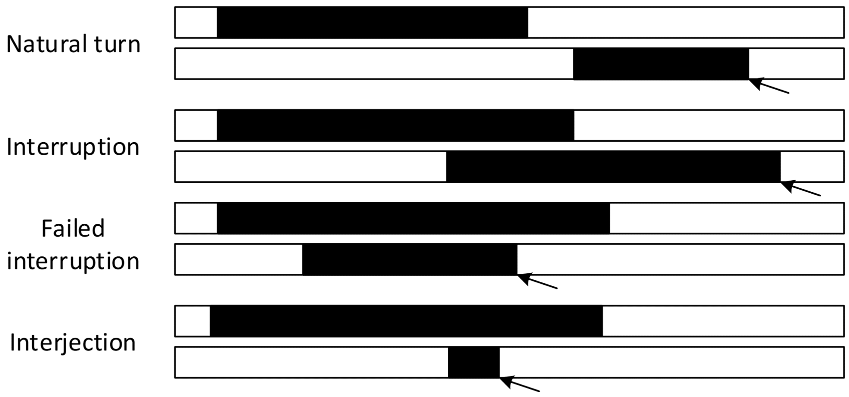

Supplementary Figure 1: Illustration of the conversational cues. There is a bar for each of the two speakers, where a black (white) area indicates that the person is speaking (silent).

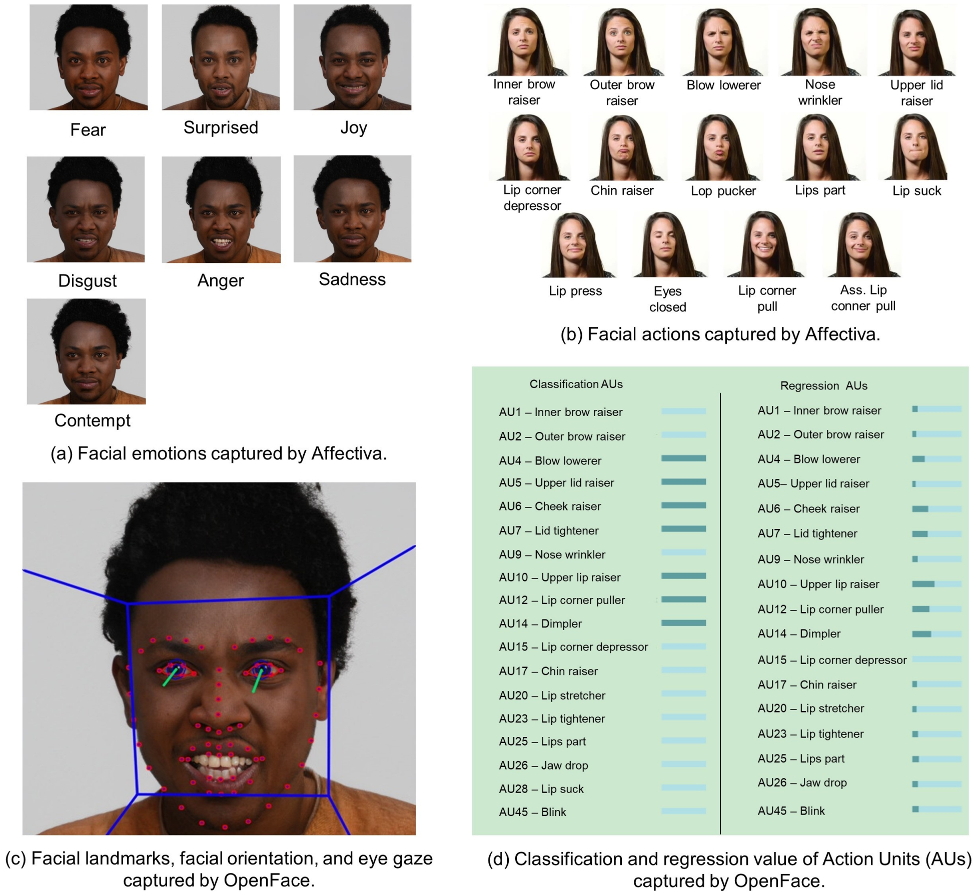

(a) Facial emotions captured by Affectiva.

(b) Facial actions captured by Affectiva.

(c) Facial landmarks, facial orientation, and eye gaze captured by OpenFace.

(d) Classification and regression value of Action Units (AUs) captured by OpenFace.

Supplementary Figure 2: Facial expressions captured by Affectiva[50] and OpenFace[51] toolkits.

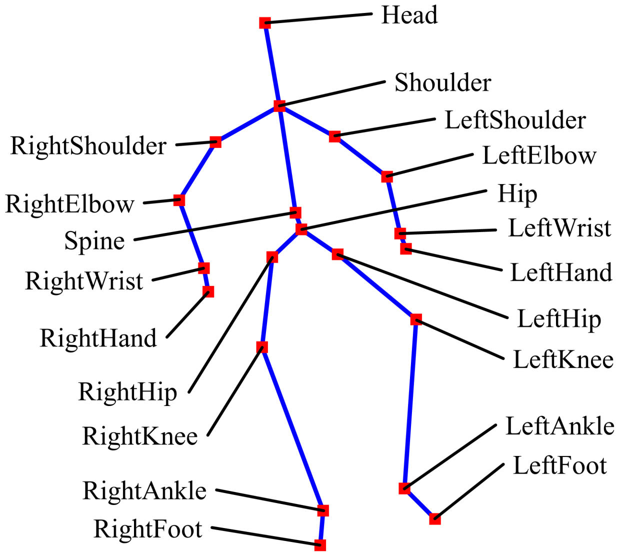

Supplementary Figure 3: Body joints captured by Microsoft Kinect.

References

[1] Keefe, R. The Brief Assessment of Cognition in Schizophrenia: reliability, sensitivity, and comparison with a standard neurocognitive battery. *Schizophrenia Research* **68**, 283–297 (2004).

[2] Yang, Z., Lim, K., Lam, M., Keefe, R. & Lee, J. Factor structure of the positive and negative syndrome scale (PANSS) in people at ultra high risk (UHR) for psychosis. *Schizophrenia Research* **201**, 85–90 (2018).

[3] Leucht, S. *et al.* Linking the PANSS, BPRS, and CGI: Clinical Implications. *Neuropsychopharmacology* **31**, 2318–2325 (2006).

[4] Leucht, S. *et al.* What does the PANSS mean? *Schizophrenia Research* **79**, 231–238 (2005).

[5] Leucht, S. *et al.* Linking PANSS negative symptom scores with the Clinical Global Impressions Scale: understanding negative symptom scores in schizophrenia. *Neuropsychopharmacology* **44**, 1589–1596 (2019).

[6] Xu, S. *et al.* Automatic Verbal Analysis of Interviews with Schizophrenic Patients. In *2018 IEEE 23rd International Conference on Digital Signal Processing (DSP)*, 1–5 (IEEE, Shanghai, China, 2018).

[7] Cohen, A. S. *et al.* Using machine learning of computerized vocal expression to measure blunted vocal affect and alogia. *npj Schizophrenia* **6**, 1–9 (2020). Number: 1 Publisher: Nature Publishing Group.

[8] Chakraborty, D. *et al.* Prediction of Negative Symptoms of Schizophrenia from Emotion Related Low-Level Speech Signals. In *2018 IEEE International Conference on Acoustics, Speech and Signal Processing (ICASSP)*, 6024–6028 (2018).

[9] Wörtwein, T. *et al.* Computational Analysis of Acoustic Descriptors in Psychotic Patients. In *Interspeech 2017*, 3256–3260 (ISCA, 2017).

[10] Bishay, M., Priebe, S. & Patras, I. Can Automatic Facial Expression Analysis Be Used for Treatment Outcome Estimation in Schizophrenia? In *ICASSP 2019 - 2019 IEEE International Conference on Acoustics, Speech and Signal Processing (ICASSP)*, 1632–1636 (IEEE, Brighton, United Kingdom, 2019).

[11] Tron, T., Peled, A., Grinsphoon, A. & Weinshall, D. Facial expressions and flat affect in schizophrenia, automatic analysis from depth camera data. In *2016 IEEE-EMBS International Conference on Biomedical and Health Informatics (BHI)*, 220–223 (2016).

[12] Tron, T., Peled, A., Grinsphoon, A. & Weinshall, D. Automated Facial Expressions Analysis in Schizophrenia: A Continuous Dynamic Approach. In *Pervasive Computing Paradigms for Mental Health*, 72–81 (Springer, Cham, 2015).

[13] Chakraborty, D. *et al.* Assessment and prediction of negative symptoms of schizophrenia from RGB+D movement signals. In *2017 IEEE 19th International Workshop on Multimedia Signal Processing (MMSP)*, 1–6 (2017).

[14] Xu, S. *et al.* Automated Verbal and Non-verbal Speech Analysis of Interviews of Individuals with Schizophrenia and Depression. In *2019 41st Annual International Conference of the IEEE Engineering in Medicine and Biology Society (EMBC)*, 225–228 (2019).

[15] Hong, K. *et al.* Lexical use in emotional autobiographical narratives of persons with schizophrenia and healthy controls. *Psychiatry Research* **225**, 40–49 (2015).

[16] Elvevåg, B., Foltz, P. W., Weinberger, D. R. & Goldberg, T. E. Quantifying incoherence in speech: An automated methodology and novel application to schizophrenia. *Schizophrenia research* **93**, 304–316 (2007).

[17] Elvevåg, B., Foltz, P. W., Rosenstein, M. & DeLisi, L. E. An automated method to analyze language use in patients with schizophrenia and their first-degree relatives. *Journal of Neurolinguistics* **23**, 270–284 (2010).

[18] Corcoran, C. M. *et al.* Prediction of psychosis across protocols and risk cohorts using automated language analysis. *World Psychiatry* **17**, 67–75 (2018).

[19] Bar, K. *et al.* Semantic Characteristics of Schizophrenic Speech. In *Proceedings of the Sixth Workshop on Computational Linguistics and Clinical Psychology*, 84–93 (Association for Computational Linguistics, Minneapolis, Minnesota, 2019).

[20] Mota, N. B. *et al.* Speech Graphs Provide a Quantitative Measure of Thought Disorder in Psychosis. *PLoS ONE* **7**, e34928 (2012).

[21] Rezaii, N., Walker, E. & Wolff, P. A machine learning approach to predicting psychosis using semantic density and latent content analysis. *npj Schizophrenia* **5**, 9 (2019).

[22] Xu, S. *et al.* Automated Lexical Analysis of Interviews with Individuals with Schizophrenia. In D'Haro, L. F., Banchs, R. E. & Li, H. (eds.) *9th International Workshop on Spoken Dialogue System Technology*, 185–197 (Springer Singapore, Singapore, 2019).

[23] Voppel, A., de Boer, J., Brederoo, S., Schnack, H. & Sommer, I. Quantified language connectedness in schizophrenia-spectrum disorders. *Psychiatry Research* **304**, 114130 (2021).

[24] Tang, S. X. *et al.* Natural language processing methods are sensitive to sub-clinical linguistic differences in schizophrenia spectrum disorders. *npj Schizophrenia* **7**, 1–8 (2021). Number: 1 Publisher: Nature Publishing Group.

[25] Kliper, R., Vaizman, Y., Weinshall, D. & Portuguese, S. Evidence for depression and schizophrenia in speech prosody. 85–88 (2010).

[26] Tahir, Y. *et al.* Non-verbal speech analysis of interviews with schizophrenic patients. In *2016 IEEE International Conference on Acoustics, Speech and Signal Processing (ICASSP)*, 5810–5814 (2016).

[27] Martínez-Sánchez, F. *et al.* Can the Acoustic Analysis of Expressive Prosody Discriminate Schizophrenia? *The Spanish Journal of Psychology* **18**, E86 (2015).

[28] Rapcan, V. *et al.* Acoustic and temporal analysis of speech: A potential biomarker for schizophrenia. *Medical Engineering & Physics* **32**, 1074–1079 (2010).

[29] Qureshi, S. A., Dias, G., Hasanuzzaman, M. & Saha, S. Improving Depression Level Estimation by Concurrently Learning Emotion Intensity. *IEEE Computational Intelligence Magazine* **15**, 47–59 (2020).

[30] Scherer, S. *et al.* Automatic behavior descriptors for psychological disorder analysis. In *2013 10th IEEE International Conference and Workshops on Automatic Face and Gesture Recognition (FG)*, 1–8 (2013).

[31] Harati, A. *et al.* Speech-Based Depression Prediction Using Encoder-Weight-Only Transfer Learning and a Large Corpus. In *ICASSP 2021 - 2021 IEEE International Conference on Acoustics, Speech and Signal Processing (ICASSP)*, 7273–7277 (2021).

[32] Espinola, C. W., Gomes, J. C., Pereira, J. M. S. & dos Santos, W. P. Detection of major depressive disorder using vocal acoustic analysis and machine learning—an exploratory study. *Research on Biomedical Engineering* **37**, 53–64 (2021).

[33] Low, L.-S. A., Maddage, M. C., Lech, M., Sheeber, L. B. & Allen, N. B. Detection of Clinical Depression in Adolescents' Speech During Family Interactions. *IEEE Transactions on Biomedical Engineering* **58**, 574–586 (2011).

[34] Ooi, K. E. B., Lech, M. & Allen, N. B. Multichannel Weighted Speech Classification System for Prediction of Major Depression in Adolescents. *IEEE Transactions on Biomedical Engineering* **60**, 497–506 (2013).

[35] Huang, Z. *et al.* Domain Adaptation for Enhancing Speech-Based Depression Detection in Natural Environmental Conditions Using Dilated CNNs. In *Interspeech 2020*, 4561–4565 (ISCA, 2020).

[36] Sanchez, M. H. *et al.* Using prosodic and spectral features in detecting depression in elderly males. In *12th Annual Conference of the International Speech Communication Association*, 3001–3004 (2011).

[37] Taguchi, T. Major depressive disorder discrimination using vocal acoustic features. *Journal of Affective Disorders* 7 (2018).

[38] Dibeklioğlu, H., Hammal, Z. & Cohn, J. F. Dynamic Multimodal Measurement of Depression Severity Using Deep Autoencoding. *IEEE Journal of Biomedical and Health Informatics* **22**, 525–536 (2018).

[39] Cohn, J. F. *et al.* Detecting depression from facial actions and vocal prosody. In *2009 3rd International Conference on Affective Computing and Intelligent Interaction and Workshops*, 1–7 (IEEE, Amsterdam, Netherlands, 2009).

[40] Horigome, T. *et al.* Evaluating the severity of depressive symptoms using upper body motion captured by RGB-depth sensors and machine learning in a clinical interview setting: A preliminary study. *Comprehensive Psychiatry* **98**, 152169 (2020).

[41] Joshi, J., Goecke, R., Parker, G. & Breakspear, M. Can body expressions contribute to automatic depression analysis? In *2013 10th IEEE International Conference and Workshops on Automatic Face and Gesture Recognition (FG)*, 1–7 (2013).

[42] Lu, Y. *et al.* Robust Speech and Natural Language Processing Models for Depression Screening. In *2020 IEEE Signal Processing in Medicine and Biology Symposium (SPMB)*, 1–5 (2020).

[43] Yang, L. *et al.* Decision Tree Based Depression Classification from Audio Video and Language Information. In *Proceedings of the 6th International Workshop on Audio/Visual Emotion Challenge*, AVEC '16, 89–96 (Association for Computing Machinery, New York, NY, USA, 2016).

[44] Alghowinem, S. *et al.* Cross-cultural detection of depression from nonverbal behaviour. In *2015 11th IEEE International Conference and Workshops on Automatic Face and Gesture Recognition (FG)*, vol. 1, 1–8 (2015).

[45] Dibeklioğlu, H., Hammal, Z., Yang, Y. & Cohn, J. F. Multimodal Detection of Depression in Clinical Interviews. In *Proceedings of the 2015 ACM on International Conference on Multimodal Interaction*, ICMI '15, 307–310 (ACM, New York, NY, USA, 2015).

[46] Valstar, M. *et al.* AVEC 2016 - Depression, Mood, and Emotion Recognition Workshop and Challenge. *arXiv:1605.01600 [cs]* (2016).

[47] Alghowinem, S. *et al.* Multimodal Depression Detection: Fusion Analysis of Paralinguistic, Head Pose and Eye Gaze Behaviors. *IEEE Transactions on Affective Computing* **9**, 478–490 (2018).

[48] Joshi, J. *et al.* Multimodal assistive technologies for depression diagnosis and monitoring. *Journal on Multimodal User Interfaces* **7**, 217–228 (2013).

[49] Lott, P. R., Guggenbühl, S., Schneeberger, A., Pulver, A. E. & Stassen, H. H. Linguistic Analysis of the Speech Output of Schizophrenic, Bipolar, and Depressive Patients. *Psychopathology* **35**, 220–227 (2002).

[50] McDuff, D. *et al.* Affectiva-MIT Facial Expression Dataset (AM-FED): Naturalistic and Spontaneous Facial Expressions Collected "In-the-Wild". In *2013 IEEE Conference on Computer Vision and Pattern Recognition Workshops*, 881–888 (2013).

[51] Baltrusaitis, T., Zadeh, A., Lim, Y. C. & Morency, L.-P. OpenFace 2.0: Facial Behavior Analysis Toolkit. In *2018 13th IEEE International Conference on Automatic Face Gesture Recognition (FG 2018)*, 59–66 (2018).
